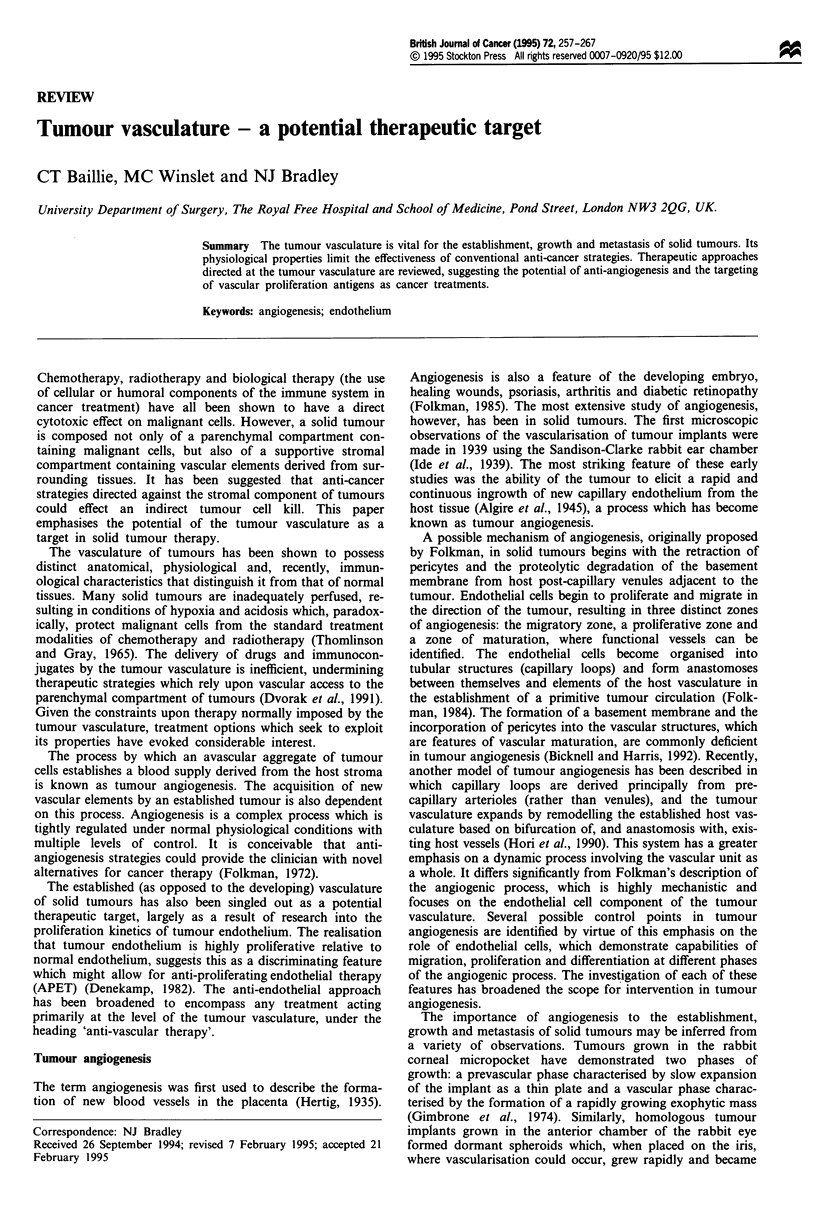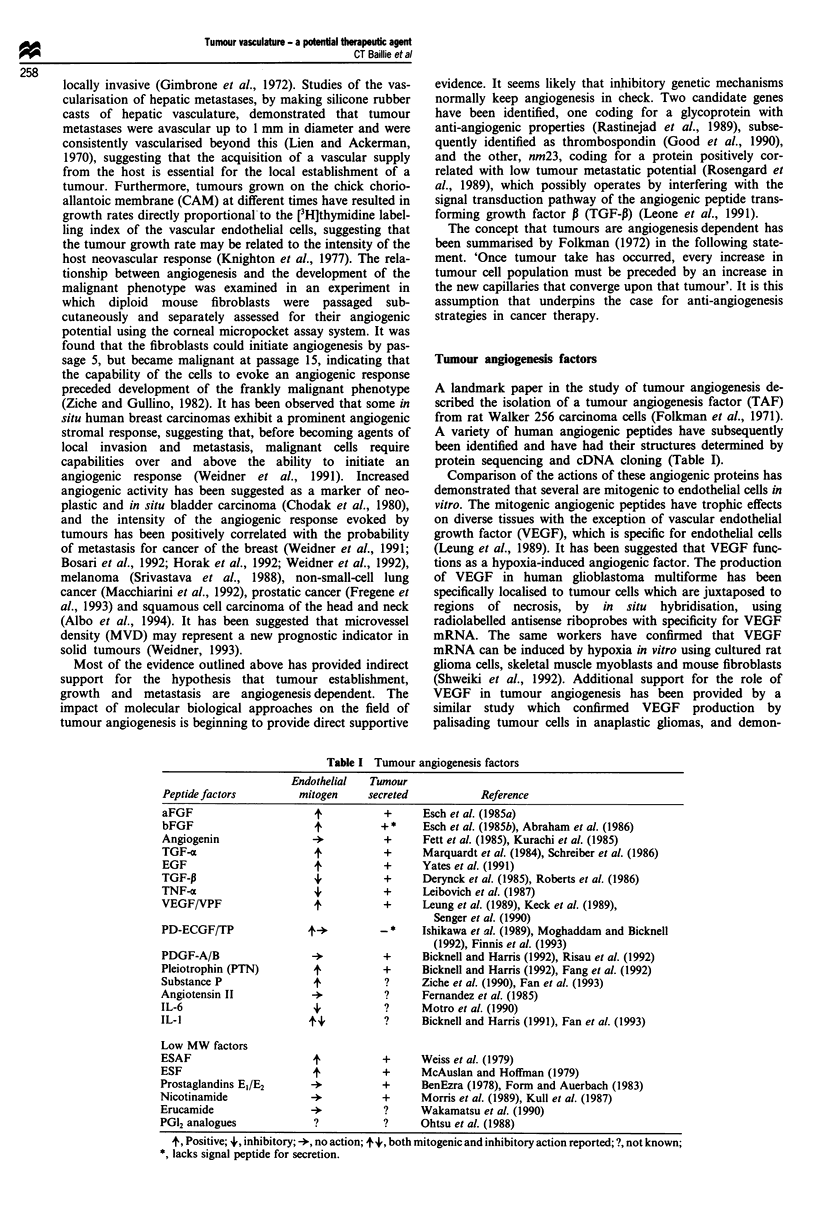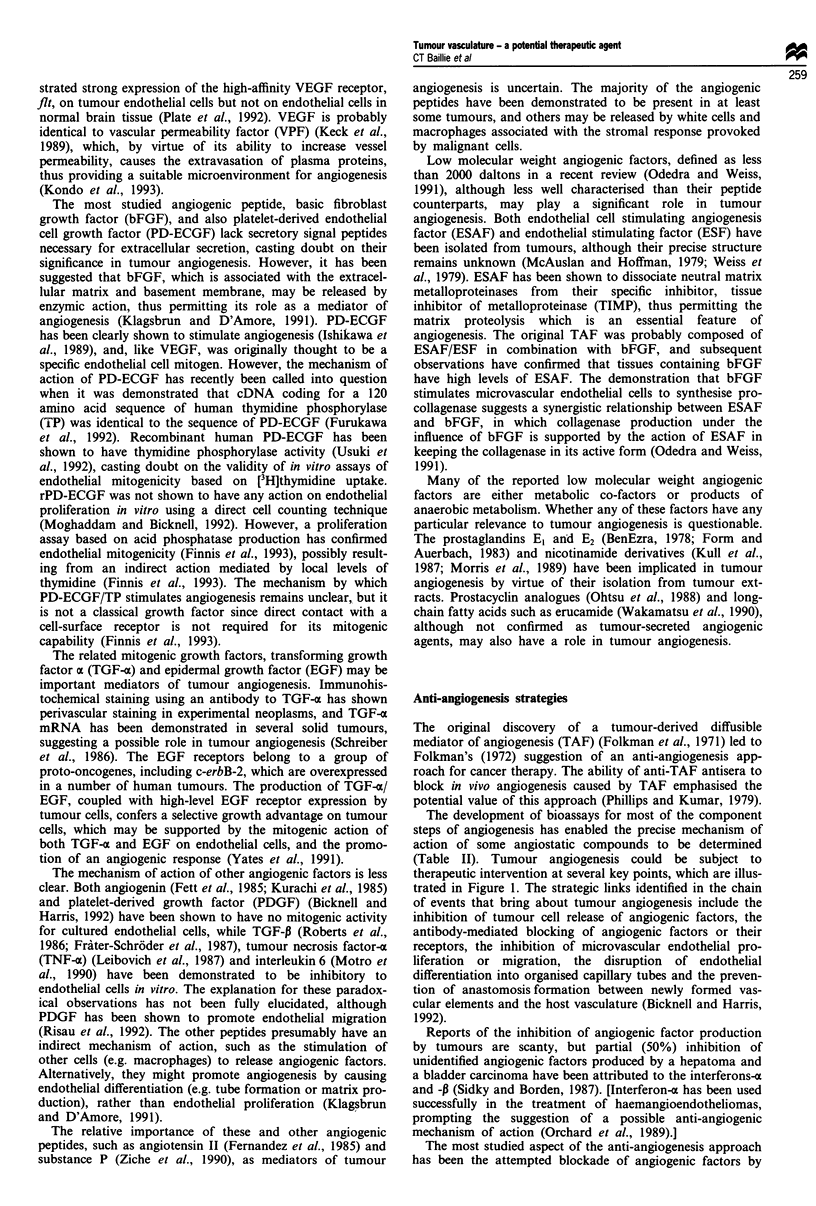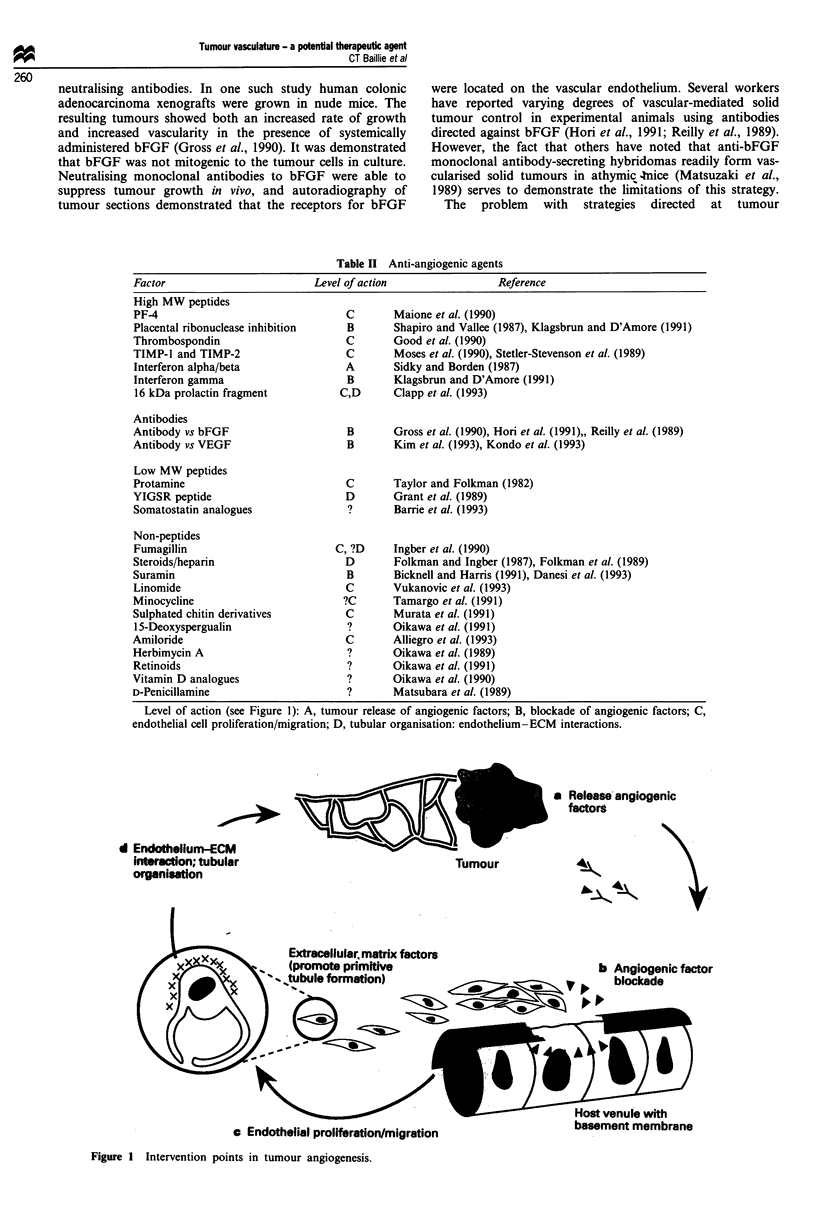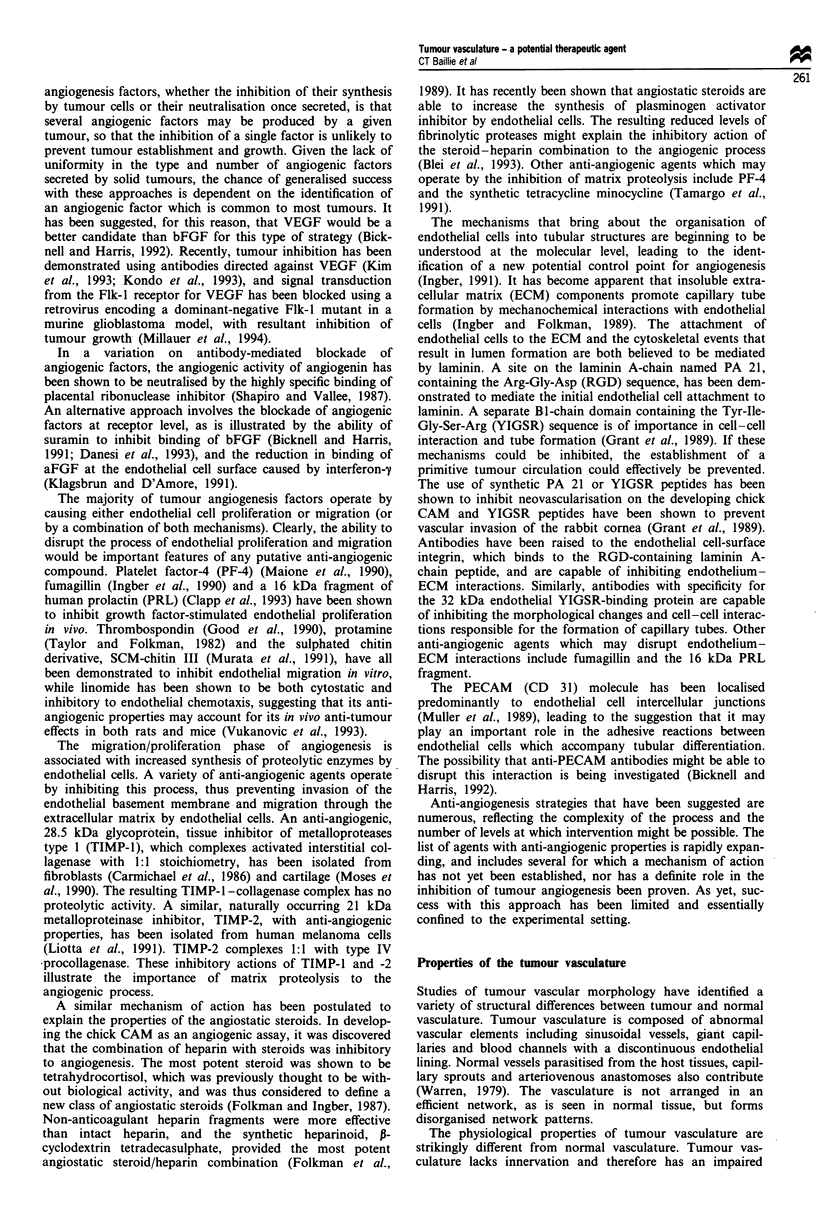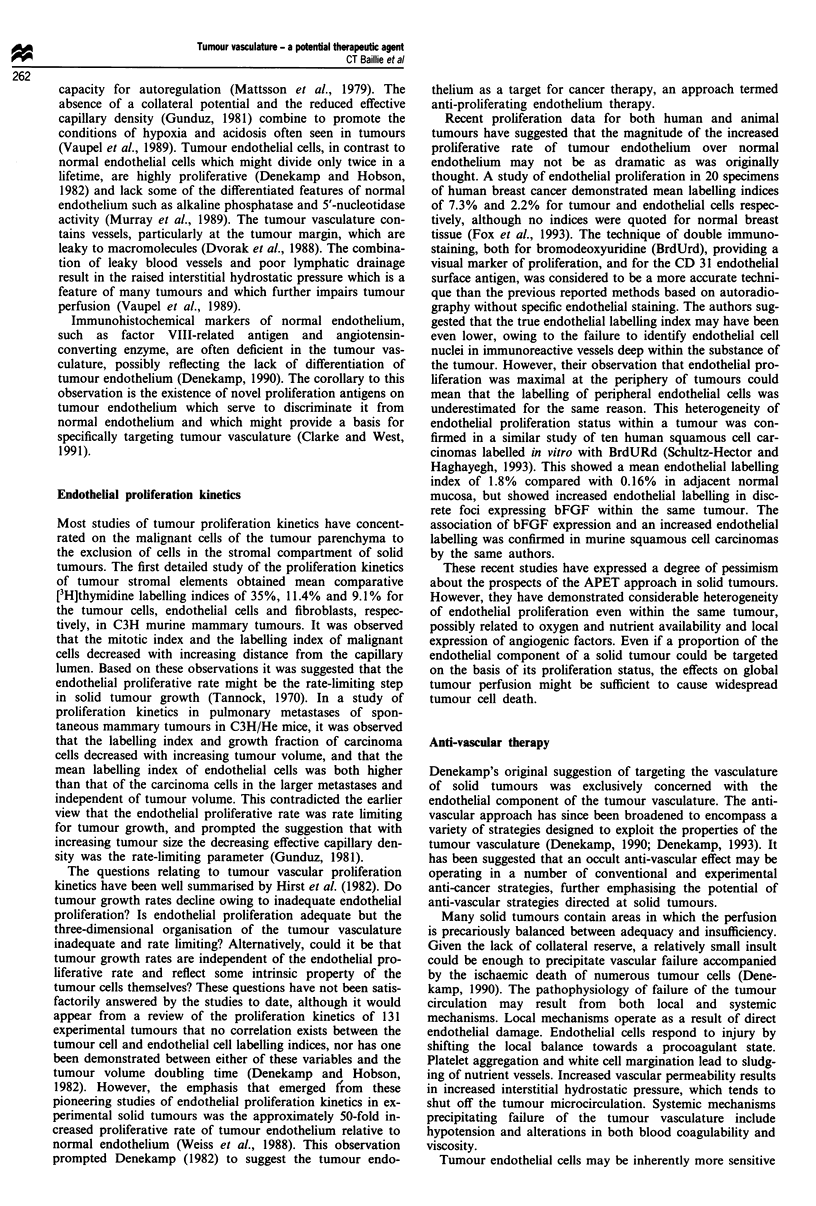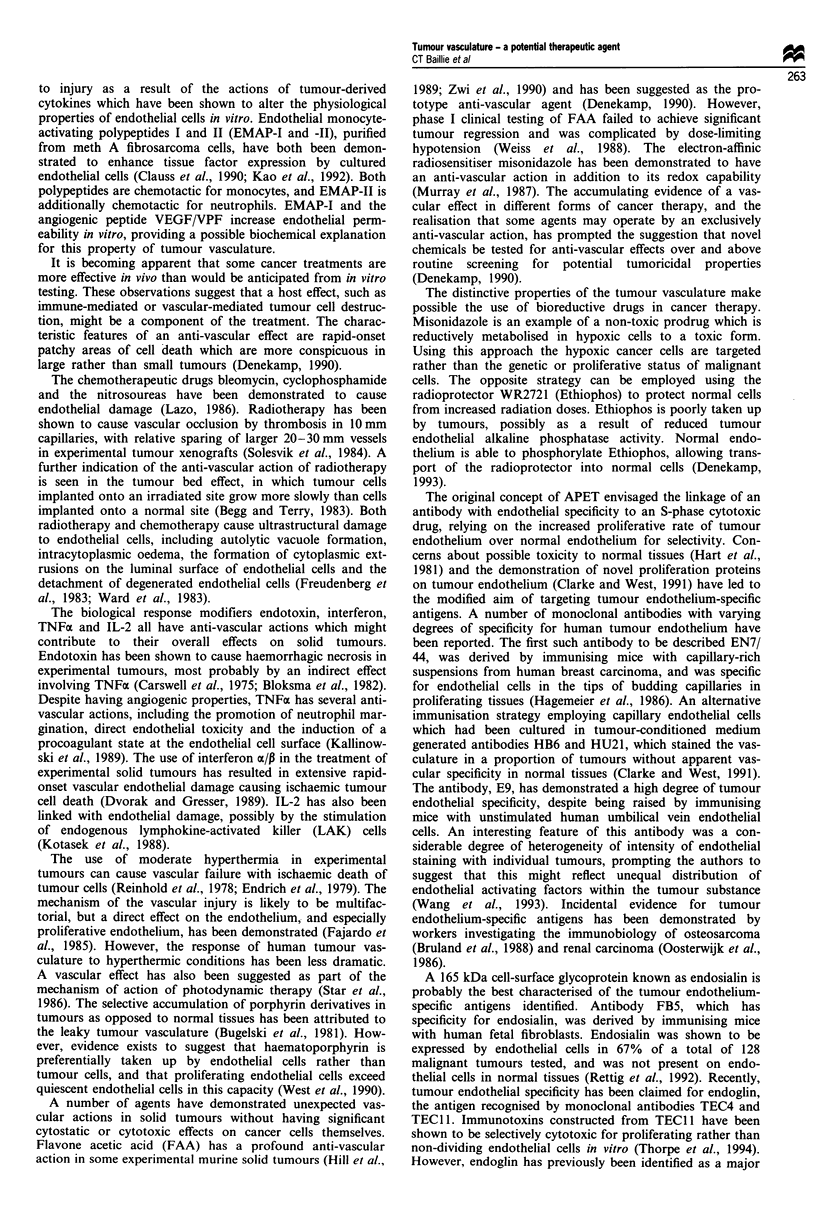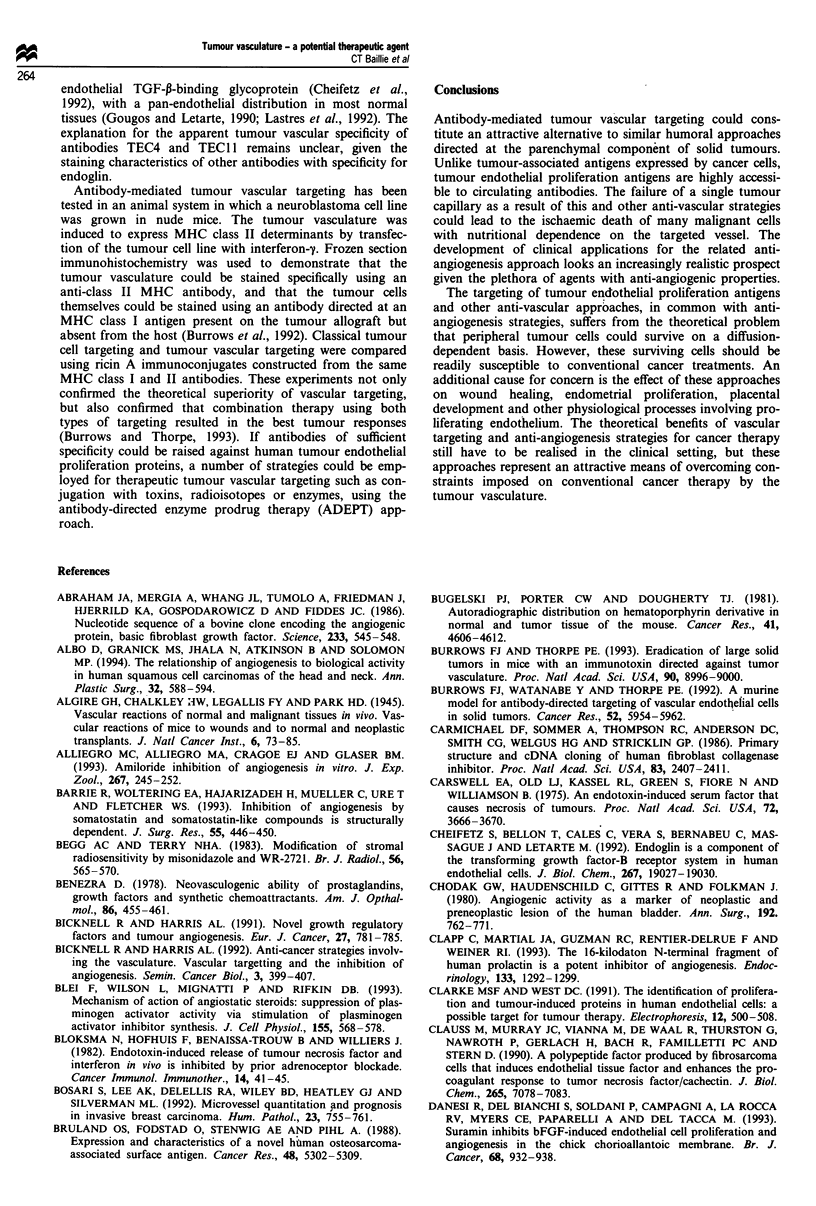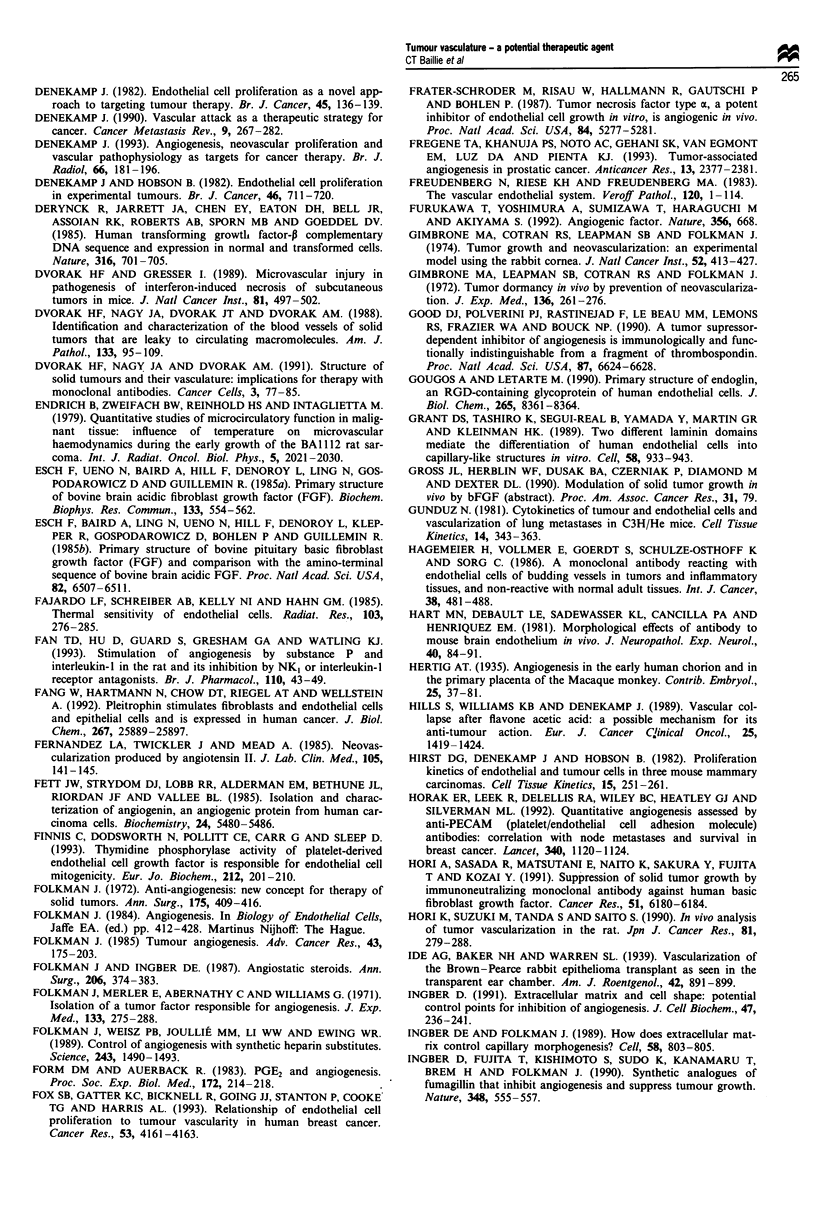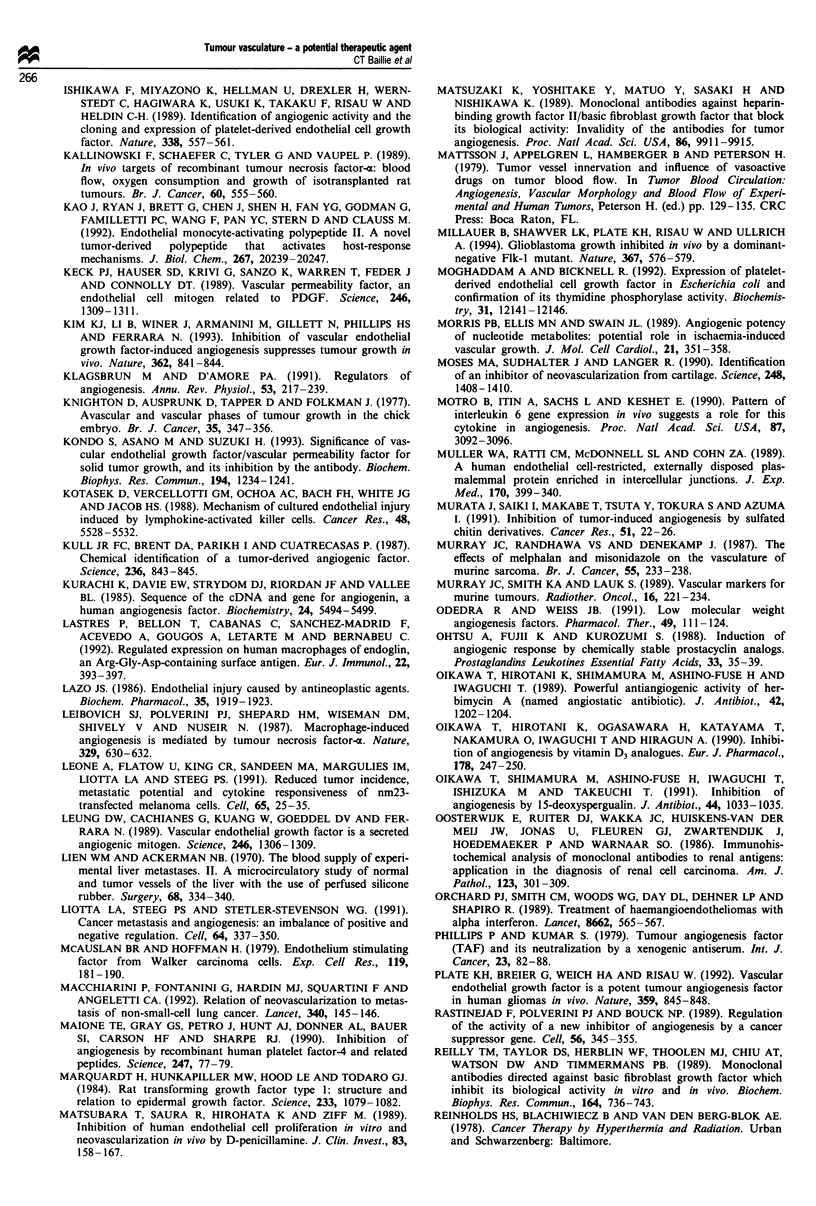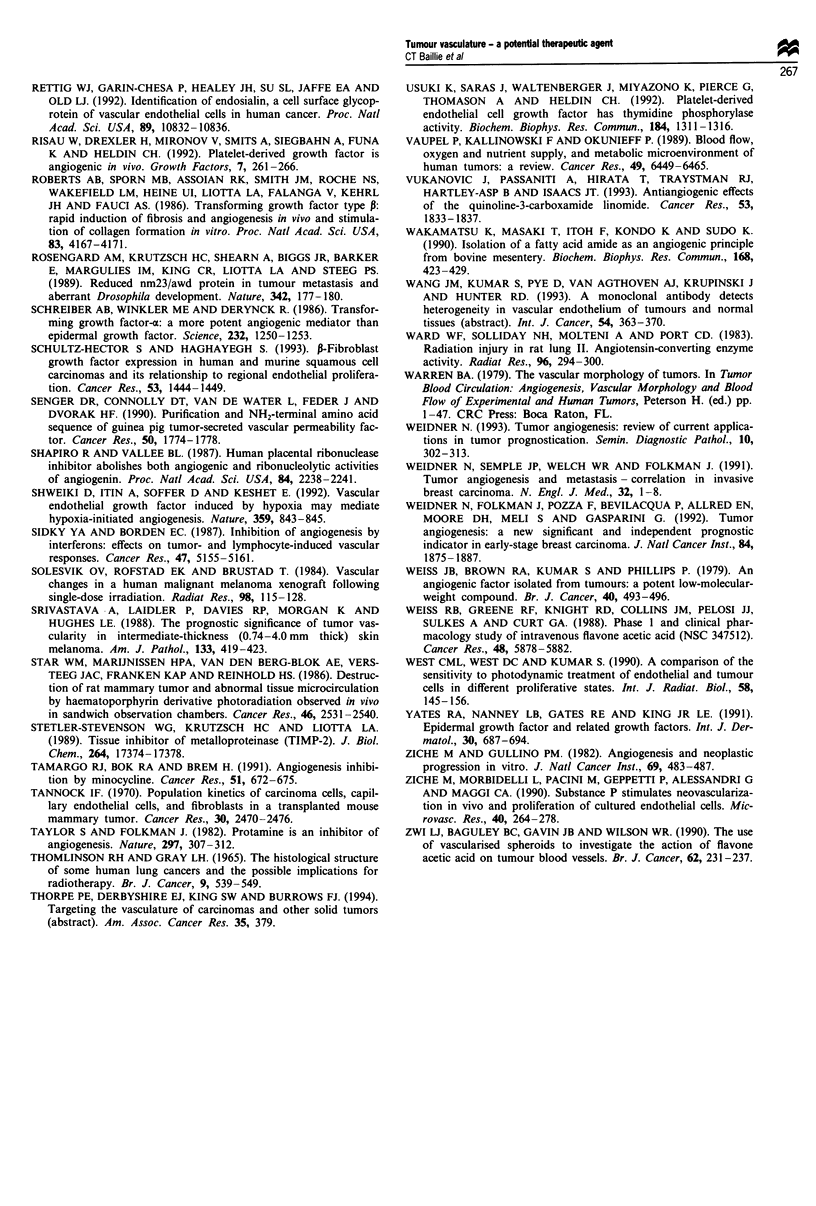# Tumour vasculature--a potential therapeutic target.

**DOI:** 10.1038/bjc.1995.323

**Published:** 1995-08

**Authors:** C. T. Baillie, M. C. Winslet, N. J. Bradley

**Affiliations:** University Department of Surgery, Royal Free Hospital and School of Medicine, London, UK.

## Abstract

The tumour vasculature is vital for the establishment, growth and metastasis of solid tumours. Its physiological properties limit the effectiveness of conventional anti-cancer strategies. Therapeutic approaches directed at the tumour vasculature are reviewed, suggesting the potential of anti-angiogenesis and the targeting of vascular proliferation antigens as cancer treatments.


British Journal of Cancer (1995) 72, 257-267
© 1995 Stockton Press All rights reserved 0007-0920/95 $12.00

REVIEW

### Tumour vasculature – a potential therapeutic target

CT Baillie, MC Winslet and NJ Bradley

*University Department of Surgery, The Royal Free Hospital and School of Medicine, Pond Street, London NW3 2QG, UK.*

**Summary** The tumour vasculature is vital for the establishment, growth and metastasis of solid tumours. Its physiological properties limit the effectiveness of conventional anti-cancer strategies. Therapeutic approaches directed at the tumour vasculature are reviewed, suggesting the potential of anti-angiogenesis and the targeting of vascular proliferation antigens as cancer treatments.

**Keywords:** angiogenesis; endothelium

Chemotherapy, radiotherapy and biological therapy (the use of cellular or humoral components of the immune system in cancer treatment) have all been shown to have a direct cytotoxic effect on malignant cells. However, a solid tumour is composed not only of a parenchymal compartment containing malignant cells, but also of a supportive stromal compartment containing vascular elements derived from surrounding tissues. It has been suggested that anti-cancer strategies directed against the stromal component of tumours could effect an indirect tumour cell kill. This paper emphasises the potential of the tumour vasculature as a target in solid tumour therapy.

The vasculature of tumours has been shown to possess distinct anatomical, physiological and, recently, immunological characteristics that distinguish it from that of normal tissues. Many solid tumours are inadequately perfused, resulting in conditions of hypoxia and acidosis which, paradoxically, protect malignant cells from the standard treatment modalities of chemotherapy and radiotherapy (Thomlinson and Gray, 1965). The delivery of drugs and immunoconjugates by the tumour vasculature is inefficient, undermining therapeutic strategies which rely upon vascular access to the parenchymal compartment of tumours (Dvorak *et al.*, 1991). Given the constraints upon therapy normally imposed by the tumour vasculature, treatment options which seek to exploit its properties have evoked considerable interest.

The process by which an avascular aggregate of tumour cells establishes a blood supply derived from the host stroma is known as tumour angiogenesis. The acquisition of new vascular elements by an established tumour is also dependent on this process. Angiogenesis is a complex process which is tightly regulated under normal physiological conditions with multiple levels of control. It is conceivable that anti-angiogenesis strategies could provide the clinician with novel alternatives for cancer therapy (Folkman, 1972).

The established (as opposed to the developing) vasculature of solid tumours has also been singled out as a potential therapeutic target, largely as a result of research into the proliferation kinetics of tumour endothelium. The realisation that tumour endothelium is highly proliferative relative to normal endothelium, suggests this as a discriminating feature which might allow for anti-proliferating endothelial therapy (APET) (Denekamp, 1982). The anti-endothelial approach has been broadened to encompass any treatment acting primarily at the level of the tumour vasculature, under the heading 'anti-vascular therapy'.

### Tumour angiogenesis

The term angiogenesis was first used to describe the formation of new blood vessels in the placenta (Hertig, 1935). Angiogenesis is also a feature of the developing embryo, healing wounds, psoriasis, arthritis and diabetic retinopathy (Folkman, 1985). The most extensive study of angiogenesis, however, has been in solid tumours. The first microscopic observations of the vascularisation of tumour implants were made in 1939 using the Sandison-Clarke rabbit ear chamber (Ide *et al.*, 1939). The most striking feature of these early studies was the ability of the tumour to elicit a rapid and continuous ingrowth of new capillary endothelium from the host tissue (Algire *et al.*, 1945), a process which has become known as tumour angiogenesis.

A possible mechanism of angiogenesis, originally proposed by Folkman, in solid tumours begins with the retraction of pericytes and the proteolytic degradation of the basement membrane from host post-capillary venules adjacent to the tumour. Endothelial cells begin to proliferate and migrate in the direction of the tumour, resulting in three distinct zones of angiogenesis: the migratory zone, a proliferative zone and a zone of maturation, where functional vessels can be identified. The endothelial cells become organised into tubular structures (capillary loops) and form anastomoses between themselves and elements of the host vasculature in the establishment of a primitive tumour circulation (Folkman, 1984). The formation of a basement membrane and the incorporation of pericytes into the vascular structures, which are features of vascular maturation, are commonly deficient in tumour angiogenesis (Bicknell and Harris, 1992). Recently, another model of tumour angiogenesis has been described in which capillary loops are derived principally from pre-capillary arterioles (rather than venules), and the tumour vasculature expands by remodelling the established host vasculature based on bifurcation of, and anastomosis with, existing host vessels (Hori *et al.*, 1990). This system has a greater emphasis on a dynamic process involving the vascular unit as a whole. It differs significantly from Folkman's description of the angiogenic process, which is highly mechanistic and focuses on the endothelial cell component of the tumour vasculature. Several possible control points in tumour angiogenesis are identified by virtue of this emphasis on the role of endothelial cells, which demonstrate capabilities of migration, proliferation and differentiation at different phases of the angiogenic process. The investigation of each of these features has broadened the scope for intervention in tumour angiogenesis.

The importance of angiogenesis to the establishment, growth and metastasis of solid tumours may be inferred from a variety of observations. Tumours grown in the rabbit corneal micropocket have demonstrated two phases of growth: a prevascular phase characterised by slow expansion of the implant as a thin plate and a vascular phase characterised by the formation of a rapidly growing exophytic mass (Gimbrone *et al.*, 1974). Similarly, homologous tumour implants grown in the anterior chamber of the rabbit eye formed dormant spheroids which, when placed on the iris, where vascularisation could occur, grew rapidly and became

Correspondence: NJ Bradley
Received 26 September 1994; revised 7 February 1995; accepted 21 February 1995

locally invasive (Gimbrone *et al.*, 1972). Studies of the vascularisation of hepatic metastases, by making silicone rubber casts of hepatic vasculature, demonstrated that tumour metastases were avascular up to 1 mm in diameter and were consistently vascularised beyond this (Lien and Ackerman, 1970), suggesting that the acquisition of a vascular supply from the host is essential for the local establishment of a tumour. Furthermore, tumours grown on the chick chorio-allantoic membrane (CAM) at different times have resulted in growth rates directly proportional to the $[^3H]$thymidine labelling index of the vascular endothelial cells, suggesting that the tumour growth rate may be related to the intensity of the host neovascular response (Knighton *et al.*, 1977). The relationship between angiogenesis and the development of the malignant phenotype was examined in an experiment in which diploid mouse fibroblasts were passaged subcutaneously and separately assessed for their angiogenic potential using the corneal micropocket assay system. It was found that the fibroblasts could initiate angiogenesis by passage 5, but became malignant at passage 15, indicating that the capability of the cells to evoke an angiogenic response preceded development of the frankly malignant phenotype (Ziche and Gullino, 1982). It has been observed that some *in situ* human breast carcinomas exhibit a prominent angiogenic stromal response, suggesting that, before becoming agents of local invasion and metastasis, malignant cells require capabilities over and above the ability to initiate an angiogenic response (Weidner *et al.*, 1991). Increased angiogenic activity has been suggested as a marker of neoplastic and *in situ* bladder carcinoma (Chodak *et al.*, 1980), and the intensity of the angiogenic response evoked by tumours has been positively correlated with the probability of metastasis for cancer of the breast (Weidner *et al.*, 1991; Bosari *et al.*, 1992; Horak *et al.*, 1992; Weidner *et al.*, 1992), melanoma (Srivastava *et al.*, 1988), non-small-cell lung cancer (Macchiarini *et al.*, 1992), prostatic cancer (Fregene *et al.*, 1993) and squamous cell carcinoma of the head and neck (Albo *et al.*, 1994). It has been suggested that microvessel density (MVD) may represent a new prognostic indicator in solid tumours (Weidner, 1993).

Most of the evidence outlined above has provided indirect support for the hypothesis that tumour establishment, growth and metastasis are angiogenesis dependent. The impact of molecular biological approaches on the field of tumour angiogenesis is beginning to provide direct supportive evidence. It seems likely that inhibitory genetic mechanisms normally keep angiogenesis in check. Two candidate genes have been identified, one coding for a glycoprotein with anti-angiogenic properties (Rastinejad *et al.*, 1989), subsequently identified as thrombospondin (Good *et al.*, 1990), and the other, *nm*23, coding for a protein positively correlated with low tumour metastatic potential (Rosengard *et al.*, 1989), which possibly operates by interfering with the signal transduction pathway of the angiogenic peptide transforming growth factor $\beta$ (TGF-$\beta$) (Leone *et al.*, 1991).

The concept that tumours are angiogenesis dependent has been summarised by Folkman (1972) in the following statement. 'Once tumour take has occurred, every increase in tumour cell population must be preceded by an increase in the new capillaries that converge upon that tumour'. It is this assumption that underpins the case for anti-angiogenesis strategies in cancer therapy.

### Tumour angiogenesis factors

A landmark paper in the study of tumour angiogenesis described the isolation of a tumour angiogenesis factor (TAF) from rat Walker 256 carcinoma cells (Folkman *et al.*, 1971). A variety of human angiogenic peptides have subsequently been identified and have had their structures determined by protein sequencing and cDNA cloning (Table I).

Comparison of the actions of these angiogenic proteins has demonstrated that several are mitogenic to endothelial cells *in vitro*. The mitogenic angiogenic peptides have trophic effects on diverse tissues with the exception of vascular endothelial growth factor (VEGF), which is specific for endothelial cells (Leung *et al.*, 1989). It has been suggested that VEGF functions as a hypoxia-induced angiogenic factor. The production of VEGF in human glioblastoma multiforme has been specifically localised to tumour cells which are juxtaposed to regions of necrosis, by *in situ* hybridisation, using radiolabelled antisense riboprobes with specificity for VEGF mRNA. The same workers have confirmed that VEGF mRNA can be induced by hypoxia *in vitro* using cultured rat glioma cells, skeletal muscle myoblasts and mouse fibroblasts (Shweiki *et al.*, 1992). Additional support for the role of VEGF in tumour angiogenesis has been provided by a similar study which confirmed VEGF production by palisading tumour cells in anaplastic gliomas, and demon-

**Table I** Tumour angiogenesis factors

| *Peptide factors* | *Endothelial mitogen* | *Tumour secreted* | *Reference* |
|---|---|---|---|
| aFGF | ↑ | + | Esch *et al.* (1985*a*) |
| bFGF | ↑ | +* | Esch *et al.* (1985*b*), Abraham *et al.* (1986) |
| Angiogenin | → | + | Fett *et al.* (1985), Kurachi *et al.* (1985) |
| TGF-α | ↑ | + | Marquardt *et al.* (1984), Schreiber *et al.* (1986) |
| EGF | ↑ | + | Yates *et al.* (1991) |
| TGF-β | ↓ | + | Derynck *et al.* (1985), Roberts *et al.* (1986) |
| TNF-α | ↓ | + | Leibovich *et al.* (1987) |
| VEGF/VPF | ↑ | + | Leung *et al.* (1989), Keck *et al.* (1989), Senger *et al.* (1990) |
| PD-ECGF/TP | ↑→ | –* | Ishikawa *et al.* (1989), Moghaddam and Bicknell (1992), Finnis *et al.* (1993) |
| PDGF-A/B | → | + | Bicknell and Harris (1992), Risau *et al.* (1992) |
| Pleiotrophin (PTN) | ↑ | + | Bicknell and Harris (1992), Fang *et al.* (1992) |
| Substance P | ↑ | ? | Ziche *et al.* (1990), Fan *et al.* (1993) |
| Angiotensin II | → | ? | Fernandez *et al.* (1985) |
| IL-6 | ↓ | ? | Motro *et al.* (1990) |
| IL-1 | ↑↓ | ? | Bicknell and Harris (1991), Fan *et al.* (1993) |
| Low MW factors | | | |
| ESAF | ↑ | + | Weiss *et al.* (1979) |
| ESF | ↑ | + | McAuslan and Hoffman (1979) |
| Prostaglandins $E_1/E_2$ | → | + | BenEzra (1978), Form and Auerbach (1983) |
| Nicotinamide | → | + | Morris *et al.* (1989), Kull *et al.* (1987) |
| Erucamide | → | ? | Wakamatsu *et al.* (1990) |
| $PGI_2$ analogues | ? | ? | Ohtsu *et al.* (1988) |

↑, Positive; ↓, inhibitory; →, no action; ↑↓, both mitogenic and inhibitory action reported; ?, not known; *, lacks signal peptide for secretion.

strated strong expression of the high-affinity VEGF receptor, *flt*, on tumour endothelial cells but not on endothelial cells in normal brain tissue (Plate *et al.*, 1992). VEGF is probably identical to vascular permeability factor (VPF) (Keck *et al.*, 1989), which, by virtue of its ability to increase vessel permeability, causes the extravasation of plasma proteins, thus providing a suitable microenvironment for angiogenesis (Kondo *et al.*, 1993).

The most studied angiogenic peptide, basic fibroblast growth factor (bFGF), and also platelet-derived endothelial cell growth factor (PD-ECGF) lack secretory signal peptides necessary for extracellular secretion, casting doubt on their significance in tumour angiogenesis. However, it has been suggested that bFGF, which is associated with the extracellular matrix and basement membrane, may be released by enzymic action, thus permitting its role as a mediator of angiogenesis (Klagsbrun and D'Amore, 1991). PD-ECGF has been clearly shown to stimulate angiogenesis (Ishikawa *et al.*, 1989), and, like VEGF, was originally thought to be a specific endothelial cell mitogen. However, the mechanism of action of PD-ECGF has recently been called into question when it was demonstrated that cDNA coding for a 120 amino acid sequence of human thymidine phosphorylase (TP) was identical to the sequence of PD-ECGF (Furukawa *et al.*, 1992). Recombinant human PD-ECGF has been shown to have thymidine phosphorylase activity (Usuki *et al.*, 1992), casting doubt on the validity of *in vitro* assays of endothelial mitogenicity based on [$^3$H]thymidine uptake. rPD-ECGF was not shown to have any action on endothelial proliferation *in vitro* using a direct cell counting technique (Moghaddam and Bicknell, 1992). However, a proliferation assay based on acid phosphatase production has confirmed endothelial mitogenicity (Finnis *et al.*, 1993), possibly resulting from an indirect action mediated by local levels of thymidine (Finnis *et al.*, 1993). The mechanism by which PD-ECGF/TP stimulates angiogenesis remains unclear, but it is not a classical growth factor since direct contact with a cell-surface receptor is not required for its mitogenic capability (Finnis *et al.*, 1993).

The related mitogenic growth factors, transforming growth factor α (TGF-α) and epidermal growth factor (EGF) may be important mediators of tumour angiogenesis. Immunohistochemical staining using an antibody to TGF-α has shown perivascular staining in experimental neoplasms, and TGF-α mRNA has been demonstrated in several solid tumours, suggesting a possible role in tumour angiogenesis (Schreiber *et al.*, 1986). The EGF receptors belong to a group of proto-oncogenes, including c-*erb*B-2, which are overexpressed in a number of human tumours. The production of TGF-α/EGF, coupled with high-level EGF receptor expression by tumour cells, confers a selective growth advantage on tumour cells, which may be supported by the mitogenic action of both TGF-α and EGF on endothelial cells, and the promotion of an angiogenic response (Yates *et al.*, 1991).

The mechanism of action of other angiogenic factors is less clear. Both angiogenin (Fett *et al.*, 1985; Kurachi *et al.*, 1985) and platelet-derived growth factor (PDGF) (Bicknell and Harris, 1992) have been shown to have no mitogenic activity for cultured endothelial cells, while TGF-β (Roberts *et al.*, 1986; Fràter-Schröder *et al.*, 1987), tumour necrosis factor-α (TNF-α) (Leibovich *et al.*, 1987) and interleukin 6 (Motro *et al.*, 1990) have been demonstrated to be inhibitory to endothelial cells *in vitro*. The explanation for these paradoxical observations has not been fully elucidated, although PDGF has been shown to promote endothelial migration (Risau *et al.*, 1992). The other peptides presumably have an indirect mechanism of action, such as the stimulation of other cells (e.g. macrophages) to release angiogenic factors. Alternatively, they might promote angiogenesis by causing endothelial differentiation (e.g. tube formation or matrix production), rather than endothelial proliferation (Klagsbrun and D'Amore, 1991).

The relative importance of these and other angiogenic peptides, such as angiotensin II (Fernandez *et al.*, 1985) and substance P (Ziche *et al.*, 1990), as mediators of tumour angiogenesis is uncertain. The majority of the angiogenic peptides have been demonstrated to be present in at least some tumours, and others may be released by white cells and macrophages associated with the stromal response provoked by malignant cells.

Low molecular weight angiogenic factors, defined as less than 2000 daltons in a recent review (Odedra and Weiss, 1991), although less well characterised than their peptide counterparts, may play a significant role in tumour angiogenesis. Both endothelial cell stimulating angiogenesis factor (ESAF) and endothelial stimulating factor (ESF) have been isolated from tumours, although their precise structure remains unknown (McAuslan and Hoffman, 1979; Weiss *et al.*, 1979). ESAF has been shown to dissociate neutral matrix metalloproteinases from their specific inhibitor, tissue inhibitor of metalloproteinase (TIMP), thus permitting the matrix proteolysis which is an essential feature of angiogenesis. The original TAF was probably composed of ESAF/ESF in combination with bFGF, and subsequent observations have confirmed that tissues containing bFGF have high levels of ESAF. The demonstration that bFGF stimulates microvascular endothelial cells to synthesise pro-collagenase suggests a synergistic relationship between ESAF and bFGF, in which collagenase production under the influence of bFGF is supported by the action of ESAF in keeping the collagenase in its active form (Odedra and Weiss, 1991).

Many of the reported low molecular weight angiogenic factors are either metabolic co-factors or products of anaerobic metabolism. Whether any of these factors have any particular relevance to tumour angiogenesis is questionable. The prostaglandins $E_1$ and $E_2$ (BenEzra, 1978; Form and Auerbach, 1983) and nicotinamide derivatives (Kull *et al.*, 1987; Morris *et al.*, 1989) have been implicated in tumour angiogenesis by virtue of their isolation from tumour extracts. Prostacyclin analogues (Ohtsu *et al.*, 1988) and long-chain fatty acids such as erucamide (Wakamatsu *et al.*, 1990), although not confirmed as tumour-secreted angiogenic agents, may also have a role in tumour angiogenesis.

### Anti-angiogenesis strategies

The original discovery of a tumour-derived diffusible mediator of angiogenesis (TAF) (Folkman *et al.*, 1971) led to Folkman's (1972) suggestion of an anti-angiogenesis approach for cancer therapy. The ability of anti-TAF antisera to block *in vivo* angiogenesis caused by TAF emphasised the potential value of this approach (Phillips and Kumar, 1979).

The development of bioassays for most of the component steps of angiogenesis has enabled the precise mechanism of action of some angiostatic compounds to be determined (Table II). Tumour angiogenesis could be subject to therapeutic intervention at several key points, which are illustrated in Figure 1. The strategic links identified in the chain of events that bring about tumour angiogenesis include the inhibition of tumour cell release of angiogenic factors, the antibody-mediated blocking of angiogenic factors or their receptors, the inhibition of microvascular endothelial proliferation or migration, the disruption of endothelial differentiation into organised capillary tubes and the prevention of anastomosis formation between newly formed vascular elements and the host vasculature (Bicknell and Harris, 1992).

Reports of the inhibition of angiogenic factor production by tumours are scanty, but partial (50%) inhibition of unidentified angiogenic factors produced by a hepatoma and a bladder carcinoma have been attributed to the interferons-α and -β (Sidky and Borden, 1987). [Interferon-α has been used successfully in the treatment of haemangioendotheliomas, prompting the suggestion of a possible anti-angiogenic mechanism of action (Orchard *et al.*, 1989).]

The most studied aspect of the anti-angiogenesis approach has been the attempted blockade of angiogenic factors by neutralising antibodies. In one such study human colonic adenocarcinoma xenografts were grown in nude mice. The resulting tumours showed both an increased rate of growth and increased vascularity in the presence of systemically administered bFGF (Gross *et al*., 1990). It was demonstrated that bFGF was not mitogenic to the tumour cells in culture. Neutralising monoclonal antibodies to bFGF were able to suppress tumour growth *in vivo*, and autoradiography of tumour sections demonstrated that the receptors for bFGF were located on the vascular endothelium. Several workers have reported varying degrees of vascular-mediated solid tumour control in experimental animals using antibodies directed against bFGF (Hori *et al*., 1991; Reilly *et al*., 1989). However, the fact that others have noted that anti-bFGF monoclonal antibody-secreting hybridomas readily form vascularised solid tumours in athymic mice (Matsuzaki *et al*., 1989) serves to demonstrate the limitations of this strategy.

The problem with strategies directed at tumour

**Table II** Anti-angiogenic agents

| *Factor* | *Level of action* | *Reference* |
|---|---|---|
| High MW peptides | | |
| PF-4 | C | Maione *et al*. (1990) |
| Placental ribonuclease inhibition | B | Shapiro and Vallee (1987), Klagsbrun and D'Amore (1991) |
| Thrombospondin | C | Good *et al*. (1990) |
| TIMP-1 and TIMP-2 | C | Moses *et al*. (1990), Stetler-Stevenson *et al*. (1989) |
| Interferon alpha/beta | A | Sidky and Borden (1987) |
| Interferon gamma | B | Klagsbrun and D'Amore (1991) |
| 16 kDa prolactin fragment | C,D | Clapp *et al*. (1993) |
| Antibodies | | |
| Antibody *vs* bFGF | B | Gross *et al*. (1990), Hori *et al*. (1991),, Reilly *et al*. (1989) |
| Antibody *vs* VEGF | B | Kim *et al*. (1993), Kondo *et al*. (1993) |
| Low MW peptides | | |
| Protamine | C | Taylor and Folkman (1982) |
| YIGSR peptide | D | Grant *et al*. (1989) |
| Somatostatin analogues | ? | Barrie *et al*. (1993) |
| Non-peptides | | |
| Fumagillin | C, ?D | Ingber *et al*. (1990) |
| Steroids/heparin | D | Folkman and Ingber (1987), Folkman *et al*. (1989) |
| Suramin | B | Bicknell and Harris (1991), Danesi *et al*. (1993) |
| Linomide | C | Vukanovic *et al*. (1993) |
| Minocycline | ?C | Tamargo *et al*. (1991) |
| Sulphated chitin derivatives | C | Murata *et al*. (1991) |
| 15-Deoxyspergualin | ? | Oikawa *et al*. (1991) |
| Amiloride | C | Alliegro *et al*. (1993) |
| Herbimycin A | ? | Oikawa *et al*. (1989) |
| Retinoids | ? | Oikawa *et al*. (1991) |
| Vitamin D analogues | ? | Oikawa *et al*. (1990) |
| D-Penicillamine | ? | Matsubara *et al*. (1989) |

Level of action (see Figure 1): A, tumour release of angiogenic factors; B, blockade of angiogenic factors; C, endothelial cell proliferation/migration; D, tubular organisation: endothelium–ECM interactions.

**Figure 1** Intervention points in tumour angiogenesis.

angiogenesis factors, whether the inhibition of their synthesis by tumour cells or their neutralisation once secreted, is that several angiogenic factors may be produced by a given tumour, so that the inhibition of a single factor is unlikely to prevent tumour establishment and growth. Given the lack of uniformity in the type and number of angiogenic factors secreted by solid tumours, the chance of generalised success with these approaches is dependent on the identification of an angiogenic factor which is common to most tumours. It has been suggested, for this reason, that VEGF would be a better candidate than bFGF for this type of strategy (Bicknell and Harris, 1992). Recently, tumour inhibition has been demonstrated using antibodies directed against VEGF (Kim *et al.*, 1993; Kondo *et al.*, 1993), and signal transduction from the Flk-1 receptor for VEGF has been blocked using a retrovirus encoding a dominant-negative Flk-1 mutant in a murine glioblastoma model, with resultant inhibition of tumour growth (Millauer *et al.*, 1994).

In a variation on antibody-mediated blockade of angiogenic factors, the angiogenic activity of angiogenin has been shown to be neutralised by the highly specific binding of placental ribonuclease inhibitor (Shapiro and Vallee, 1987). An alternative approach involves the blockade of angiogenic factors at receptor level, as is illustrated by the ability of suramin to inhibit binding of bFGF (Bicknell and Harris, 1991; Danesi *et al.*, 1993), and the reduction in binding of aFGF at the endothelial cell surface caused by interferon-γ (Klagsbrun and D'Amore, 1991).

The majority of tumour angiogenesis factors operate by causing either endothelial cell proliferation or migration (or by a combination of both mechanisms). Clearly, the ability to disrupt the process of endothelial proliferation and migration would be important features of any putative anti-angiogenic compound. Platelet factor-4 (PF-4) (Maione *et al.*, 1990), fumagillin (Ingber *et al.*, 1990) and a 16 kDa fragment of human prolactin (PRL) (Clapp *et al.*, 1993) have been shown to inhibit growth factor-stimulated endothelial proliferation *in vivo*. Thrombospondin (Good *et al.*, 1990), protamine (Taylor and Folkman, 1982) and the sulphated chitin derivative, SCM-chitin III (Murata *et al.*, 1991), have all been demonstrated to inhibit endothelial migration *in vitro*, while linomide has been shown to be both cytostatic and inhibitory to endothelial chemotaxis, suggesting that its anti-angiogenic properties may account for its *in vivo* anti-tumour effects in both rats and mice (Vukanovic *et al.*, 1993).

The migration/proliferation phase of angiogenesis is associated with increased synthesis of proteolytic enzymes by endothelial cells. A variety of anti-angiogenic agents operate by inhibiting this process, thus preventing invasion of the endothelial basement membrane and migration through the extracellular matrix by endothelial cells. An anti-angiogenic, 28.5 kDa glycoprotein, tissue inhibitor of metalloproteases type 1 (TIMP-1), which complexes activated interstitial collagenase with 1:1 stoichiometry, has been isolated from fibroblasts (Carmichael *et al.*, 1986) and cartilage (Moses *et al.*, 1990). The resulting TIMP-1-collagenase complex has no proteolytic activity. A similar, naturally occurring 21 kDa metalloproteinase inhibitor, TIMP-2, with anti-angiogenic properties, has been isolated from human melanoma cells (Liotta *et al.*, 1991). TIMP-2 complexes 1:1 with type IV procollagenase. These inhibitory actions of TIMP-1 and -2 illustrate the importance of matrix proteolysis to the angiogenic process.

A similar mechanism of action has been postulated to explain the properties of the angiostatic steroids. In developing the chick CAM as an angiogenic assay, it was discovered that the combination of heparin with steroids was inhibitory to angiogenesis. The most potent steroid was shown to be tetrahydrocortisol, which was previously thought to be without biological activity, and was thus considered to define a new class of angiostatic steroids (Folkman and Ingber, 1987). Non-anticoagulant heparin fragments were more effective than intact heparin, and the synthetic heparinoid, β-cyclodextrin tetradecasulphate, provided the most potent angiostatic steroid/heparin combination (Folkman *et al.*, 1989). It has recently been shown that angiostatic steroids are able to increase the synthesis of plasminogen activator inhibitor by endothelial cells. The resulting reduced levels of fibrinolytic proteases might explain the inhibitory action of the steroid-heparin combination to the angiogenic process (Blei *et al.*, 1993). Other anti-angiogenic agents which may operate by the inhibition of matrix proteolysis include PF-4 and the synthetic tetracycline minocycline (Tamargo *et al.*, 1991).

The mechanisms that bring about the organisation of endothelial cells into tubular structures are beginning to be understood at the molecular level, leading to the identification of a new potential control point for angiogenesis (Ingber, 1991). It has become apparent that insoluble extracellular matrix (ECM) components promote capillary tube formation by mechanochemical interactions with endothelial cells (Ingber and Folkman, 1989). The attachment of endothelial cells to the ECM and the cytoskeletal events that result in lumen formation are both believed to be mediated by laminin. A site on the laminin A-chain named PA 21, containing the Arg-Gly-Asp (RGD) sequence, has been demonstrated to mediate the initial endothelial cell attachment to laminin. A separate B1-chain domain containing the Tyr-Ile-Gly-Ser-Arg (YIGSR) sequence is of importance in cell-cell interaction and tube formation (Grant *et al.*, 1989). If these mechanisms could be inhibited, the establishment of a primitive tumour circulation could effectively be prevented. The use of synthetic PA 21 or YIGSR peptides has been shown to inhibit neovascularisation on the developing chick CAM and YIGSR peptides have been shown to prevent vascular invasion of the rabbit cornea (Grant *et al.*, 1989). Antibodies have been raised to the endothelial cell-surface integrin, which binds to the RGD-containing laminin A-chain peptide, and are capable of inhibiting endothelium-ECM interactions. Similarly, antibodies with specificity for the 32 kDa endothelial YIGSR-binding protein are capable of inhibiting the morphological changes and cell-cell interactions responsible for the formation of capillary tubes. Other anti-angiogenic agents which may disrupt endothelium-ECM interactions include fumagillin and the 16 kDa PRL fragment.

The PECAM (CD 31) molecule has been localised predominantly to endothelial cell intercellular junctions (Muller *et al.*, 1989), leading to the suggestion that it may play an important role in the adhesive reactions between endothelial cells which accompany tubular differentiation. The possibility that anti-PECAM antibodies might be able to disrupt this interaction is being investigated (Bicknell and Harris, 1992).

Anti-angiogenesis strategies that have been suggested are numerous, reflecting the complexity of the process and the number of levels at which intervention might be possible. The list of agents with anti-angiogenic properties is rapidly expanding, and includes several for which a mechanism of action has not yet been established, nor has a definite role in the inhibition of tumour angiogenesis been proven. As yet, success with this approach has been limited and essentially confined to the experimental setting.

### Properties of the tumour vasculature

Studies of tumour vascular morphology have identified a variety of structural differences between tumour and normal vasculature. Tumour vasculature is composed of abnormal vascular elements including sinusoidal vessels, giant capillaries and blood channels with a discontinuous endothelial lining. Normal vessels parasitised from the host tissues, capillary sprouts and arteriovenous anastomoses also contribute (Warren, 1979). The vasculature is not arranged in an efficient network, as is seen in normal tissue, but forms disorganised network patterns.

The physiological properties of tumour vasculature are strikingly different from normal vasculature. Tumour vasculature lacks innervation and therefore has an impaired capacity for autoregulation (Mattsson et al., 1979). The absence of a collateral potential and the reduced effective capillary density (Gunduz, 1981) combine to promote the conditions of hypoxia and acidosis often seen in tumours (Vaupel et al., 1989). Tumour endothelial cells, in contrast to normal endothelial cells which might divide only twice in a lifetime, are highly proliferative (Denekamp and Hobson, 1982) and lack some of the differentiated features of normal endothelium such as alkaline phosphatase and 5′-nucleotidase activity (Murray et al., 1989). The tumour vasculature contains vessels, particularly at the tumour margin, which are leaky to macromolecules (Dvorak et al., 1988). The combination of leaky blood vessels and poor lymphatic drainage result in the raised interstitial hydrostatic pressure which is a feature of many tumours and which further impairs tumour perfusion (Vaupel et al., 1989).

Immunohistochemical markers of normal endothelium, such as factor VIII-related antigen and angiotensin-converting enzyme, are often deficient in the tumour vasculature, possibly reflecting the lack of differentiation of tumour endothelium (Denekamp, 1990). The corollary to this observation is the existence of novel proliferation antigens on tumour endothelium which serve to discriminate it from normal endothelium and which might provide a basis for specifically targeting tumour vasculature (Clarke and West, 1991).

### Endothelial proliferation kinetics

Most studies of tumour proliferation kinetics have concentrated on the malignant cells of the tumour parenchyma to the exclusion of cells in the stromal compartment of solid tumours. The first detailed study of the proliferation kinetics of tumour stromal elements obtained mean comparative [$^3$H]thymidine labelling indices of 35%, 11.4% and 9.1% for the tumour cells, endothelial cells and fibroblasts, respectively, in C3H murine mammary tumours. It was observed that the mitotic index and the labelling index of malignant cells decreased with increasing distance from the capillary lumen. Based on these observations it was suggested that the endothelial proliferative rate might be the rate-limiting step in solid tumour growth (Tannock, 1970). In a study of proliferation kinetics in pulmonary metastases of spontaneous mammary tumours in C3H/He mice, it was observed that the labelling index and growth fraction of carcinoma cells decreased with increasing tumour volume, and that the mean labelling index of endothelial cells was both higher than that of the carcinoma cells in the larger metastases and independent of tumour volume. This contradicted the earlier view that the endothelial proliferative rate was rate limiting for tumour growth, and prompted the suggestion that with increasing tumour size the decreasing effective capillary density was the rate-limiting parameter (Gunduz, 1981).

The questions relating to tumour vascular proliferation kinetics have been well summarised by Hirst et al. (1982). Do tumour growth rates decline owing to inadequate endothelial proliferation? Is endothelial proliferation adequate but the three-dimensional organisation of the tumour vasculature inadequate and rate limiting? Alternatively, could it be that tumour growth rates are independent of the endothelial proliferative rate and reflect some intrinsic property of the tumour cells themselves? These questions have not been satisfactorily answered by the studies to date, although it would appear from a review of the proliferation kinetics of 131 experimental tumours that no correlation exists between the tumour cell and endothelial cell labelling indices, nor has one been demonstrated between either of these variables and the tumour volume doubling time (Denekamp and Hobson, 1982). However, the emphasis that emerged from these pioneering studies of endothelial proliferation kinetics in experimental solid tumours was the approximately 50-fold increased proliferative rate of tumour endothelium relative to normal endothelium (Weiss et al., 1988). This observation prompted Denekamp (1982) to suggest the tumour endothelium as a target for cancer therapy, an approach termed anti-proliferating endothelium therapy.

Recent proliferation data for both human and animal tumours have suggested that the magnitude of the increased proliferative rate of tumour endothelium over normal endothelium may not be as dramatic as was originally thought. A study of endothelial proliferation in 20 specimens of human breast cancer demonstrated mean labelling indices of 7.3% and 2.2% for tumour and endothelial cells respectively, although no indices were quoted for normal breast tissue (Fox et al., 1993). The technique of double immunostaining, both for bromodeoxyuridine (BrdUrd), providing a visual marker of proliferation, and for the CD 31 endothelial surface antigen, was considered to be a more accurate technique than the previous reported methods based on autoradiography without specific endothelial staining. The authors suggested that the true endothelial labelling index may have been even lower, owing to the failure to identify endothelial cell nuclei in immunoreactive vessels deep within the substance of the tumour. However, their observation that endothelial proliferation was maximal at the periphery of tumours could mean that the labelling of peripheral endothelial cells was underestimated for the same reason. This heterogeneity of endothelial proliferation status within a tumour was confirmed in a similar study of ten human squamous cell carcinomas labelled *in vitro* with BrdURd (Schultz-Hector and Haghayegh, 1993). This showed a mean endothelial labelling index of 1.8% compared with 0.16% in adjacent normal mucosa, but showed increased endothelial labelling in discrete foci expressing bFGF within the same tumour. The association of bFGF expression and an increased endothelial labelling was confirmed in murine squamous cell carcinomas by the same authors.

These recent studies have expressed a degree of pessimism about the prospects of the APET approach in solid tumours. However, they have demonstrated considerable heterogeneity of endothelial proliferation even within the same tumour, possibly related to oxygen and nutrient availability and local expression of angiogenic factors. Even if a proportion of the endothelial component of a solid tumour could be targeted on the basis of its proliferation status, the effects on global tumour perfusion might be sufficient to cause widespread tumour cell death.

### Anti-vascular therapy

Denekamp's original suggestion of targeting the vasculature of solid tumours was exclusively concerned with the endothelial component of the tumour vasculature. The anti-vascular approach has since been broadened to encompass a variety of strategies designed to exploit the properties of the tumour vasculature (Denekamp, 1990; Denekamp, 1993). It has been suggested that an occult anti-vascular effect may be operating in a number of conventional and experimental anti-cancer strategies, further emphasising the potential of anti-vascular strategies directed at solid tumours.

Many solid tumours contain areas in which the perfusion is precariously balanced between adequacy and insufficiency. Given the lack of collateral reserve, a relatively small insult could be enough to precipitate vascular failure accompanied by the ischaemic death of numerous tumour cells (Denekamp, 1990). The pathophysiology of failure of the tumour circulation may result from both local and systemic mechanisms. Local mechanisms operate as a result of direct endothelial damage. Endothelial cells respond to injury by shifting the local balance towards a procoagulant state. Platelet aggregation and white cell margination lead to sludging of nutrient vessels. Increased vascular permeability results in increased interstitial hydrostatic pressure, which tends to shut off the tumour microcirculation. Systemic mechanisms precipitating failure of the tumour vasculature include hypotension and alterations in both blood coagulability and viscosity.

Tumour endothelial cells may be inherently more sensitive to injury as a result of the actions of tumour-derived cytokines which have been shown to alter the physiological properties of endothelial cells *in vitro*. Endothelial monocyte-activating polypeptides I and II (EMAP-I and -II), purified from meth A fibrosarcoma cells, have both been demonstrated to enhance tissue factor expression by cultured endothelial cells (Clauss *et al*., 1990; Kao *et al*., 1992). Both polypeptides are chemotactic for monocytes, and EMAP-II is additionally chemotactic for neutrophils. EMAP-I and the angiogenic peptide VEGF/VPF increase endothelial permeability *in vitro*, providing a possible biochemical explanation for this property of tumour vasculature.

It is becoming apparent that some cancer treatments are more effective *in vivo* than would be anticipated from *in vitro* testing. These observations suggest that a host effect, such as immune-mediated or vascular-mediated tumour cell destruction, might be a component of the treatment. The characteristic features of an anti-vascular effect are rapid-onset patchy areas of cell death which are more conspicuous in large rather than small tumours (Denekamp, 1990).

The chemotherapeutic drugs bleomycin, cyclophosphamide and the nitrosoureas have been demonstrated to cause endothelial damage (Lazo, 1986). Radiotherapy has been shown to cause vascular occlusion by thrombosis in 10 mm capillaries, with relative sparing of larger 20–30 mm vessels in experimental tumour xenografts (Solesvik *et al*., 1984). A further indication of the anti-vascular action of radiotherapy is seen in the tumour bed effect, in which tumour cells implanted onto an irradiated site grow more slowly than cells implanted onto a normal site (Begg and Terry, 1983). Both radiotherapy and chemotherapy cause ultrastructural damage to endothelial cells, including autolytic vacuole formation, intracytoplasmic oedema, the formation of cytoplasmic extrusions on the luminal surface of endothelial cells and the detachment of degenerated endothelial cells (Freudenberg *et al*., 1983; Ward *et al*., 1983).

The biological response modifiers endotoxin, interferon, TNFα and IL-2 all have anti-vascular actions which might contribute to their overall effects on solid tumours. Endotoxin has been shown to cause haemorrhagic necrosis in experimental tumours, most probably by an indirect effect involving TNFα (Carswell *et al*., 1975; Bloksma *et al*., 1982). Despite having angiogenic properties, TNFα has several anti-vascular actions, including the promotion of neutrophil margination, direct endothelial toxicity and the induction of a procoagulant state at the endothelial cell surface (Kallinowski *et al*., 1989). The use of interferon α/β in the treatment of experimental solid tumours has resulted in extensive rapid-onset vascular endothelial damage causing ischaemic tumour cell death (Dvorak and Gresser, 1989). IL-2 has also been linked with endothelial damage, possibly by the stimulation of endogenous lymphokine-activated killer (LAK) cells (Kotasek *et al*., 1988).

The use of moderate hyperthermia in experimental tumours can cause vascular failure with ischaemic death of tumour cells (Reinhold *et al*., 1978; Endrich *et al*., 1979). The mechanism of the vascular injury is likely to be multifactorial, but a direct effect on the endothelium, and especially proliferative endothelium, has been demonstrated (Fajardo *et al*., 1985). However, the response of human tumour vasculature to hyperthermic conditions has been less dramatic. A vascular effect has also been suggested as part of the mechanism of action of photodynamic therapy (Star *et al*., 1986). The selective accumulation of porphyrin derivatives in tumours as opposed to normal tissues has been attributed to the leaky tumour vasculature (Bugelski *et al*., 1981). However, evidence exists to suggest that haematoporphyrin is preferentially taken up by endothelial cells rather than tumour cells, and that proliferating endothelial cells exceed quiescent endothelial cells in this capacity (West *et al*., 1990).

A number of agents have demonstrated unexpected vascular actions in solid tumours without having significant cytostatic or cytotoxic effects on cancer cells themselves. Flavone acetic acid (FAA) has a profound anti-vascular action in some experimental murine solid tumours (Hill *et al*., 1989; Zwi *et al*., 1990) and has been suggested as the prototype anti-vascular agent (Denekamp, 1990). However, phase I clinical testing of FAA failed to achieve significant tumour regression and was complicated by dose-limiting hypotension (Weiss *et al*., 1988). The electron-affinic radiosensitiser misonidazole has been demonstrated to have an anti-vascular action in addition to its redox capability (Murray *et al*., 1987). The accumulating evidence of a vascular effect in different forms of cancer therapy, and the realisation that some agents may operate by an exclusively anti-vascular action, has prompted the suggestion that novel chemicals be tested for anti-vascular effects over and above routine screening for potential tumoricidal properties (Denekamp, 1990).

The distinctive properties of the tumour vasculature make possible the use of bioreductive drugs in cancer therapy. Misonidazole is an example of a non-toxic prodrug which is reductively metabolised in hypoxic cells to a toxic form. Using this approach the hypoxic cancer cells are targeted rather than the genetic or proliferative status of malignant cells. The opposite strategy can be employed using the radioprotector WR2721 (Ethiophos) to protect normal cells from increased radiation doses. Ethiophos is poorly taken up by tumours, possibly as a result of reduced tumour endothelial alkaline phosphatase activity. Normal endothelium is able to phosphorylate Ethiophos, allowing transport of the radioprotector into normal cells (Denekamp, 1993).

The original concept of APET envisaged the linkage of an antibody with endothelial specificity to an S-phase cytotoxic drug, relying on the increased proliferative rate of tumour endothelium over normal endothelium for selectivity. Concerns about possible toxicity to normal tissues (Hart *et al*., 1981) and the demonstration of novel proliferation proteins on tumour endothelium (Clarke and West, 1991) have led to the modified aim of targeting tumour endothelium-specific antigens. A number of monoclonal antibodies with varying degrees of specificity for human tumour endothelium have been reported. The first such antibody to be described EN7/44, was derived by immunising mice with capillary-rich suspensions from human breast carcinoma, and was specific for endothelial cells in the tips of budding capillaries in proliferating tissues (Hagemeier *et al*., 1986). An alternative immunisation strategy employing capillary endothelial cells which had been cultured in tumour-conditioned medium generated antibodies HB6 and HU21, which stained the vasculature in a proportion of tumours without apparent vascular specificity in normal tissues (Clarke and West, 1991). The antibody, E9, has demonstrated a high degree of tumour endothelial specificity, despite being raised by immunising mice with unstimulated human umbilical vein endothelial cells. An interesting feature of this antibody was a considerable degree of heterogeneity of intensity of endothelial staining with individual tumours, prompting the authors to suggest that this might reflect unequal distribution of endothelial activating factors within the tumour substance (Wang *et al*., 1993). Incidental evidence for tumour endothelium-specific antigens has been demonstrated by workers investigating the immunobiology of osteosarcoma (Bruland *et al*., 1988) and renal carcinoma (Oosterwijk *et al*., 1986).

A 165 kDa cell-surface glycoprotein known as endosialin is probably the best characterised of the tumour endothelium-specific antigens identified. Antibody FB5, which has specificity for endosialin, was derived by immunising mice with human fetal fibroblasts. Endosialin was shown to be expressed by endothelial cells in 67% of a total of 128 malignant tumours tested, and was not present on endothelial cells in normal tissues (Rettig *et al*., 1992). Recently, tumour endothelial specificity has been claimed for endoglin, the antigen recognised by monoclonal antibodies TEC4 and TEC11. Immunotoxins constructed from TEC11 have been shown to be selectively cytotoxic for proliferating rather than non-dividing endothelial cells *in vitro* (Thorpe *et al*., 1994). However, endoglin has previously been identified as a major endothelial TGF-$\beta$-binding glycoprotein (Cheifetz *et al.*, 1992), with a pan-endothelial distribution in most normal tissues (Gougos and Letarte, 1990; Lastres *et al.*, 1992). The explanation for the apparent tumour vascular specificity of antibodies TEC4 and TEC11 remains unclear, given the staining characteristics of other antibodies with specificity for endoglin.

Antibody-mediated tumour vascular targeting has been tested in an animal system in which a neuroblastoma cell line was grown in nude mice. The tumour vasculature was induced to express MHC class II determinants by transfection of the tumour cell line with interferon-$\gamma$. Frozen section immunohistochemistry was used to demonstrate that the tumour vasculature could be stained specifically using an anti-class II MHC antibody, and that the tumour cells themselves could be stained using an antibody directed at an MHC class I antigen present on the tumour allograft but absent from the host (Burrows *et al.*, 1992). Classical tumour cell targeting and tumour vascular targeting were compared using ricin A immunoconjugates constructed from the same MHC class I and II antibodies. These experiments not only confirmed the theoretical superiority of vascular targeting, but also confirmed that combination therapy using both types of targeting resulted in the best tumour responses (Burrows and Thorpe, 1993). If antibodies of sufficient specificity could be raised against human tumour endothelial proliferation proteins, a number of strategies could be employed for therapeutic tumour vascular targeting such as conjugation with toxins, radioisotopes or enzymes, using the antibody-directed enzyme prodrug therapy (ADEPT) approach.

### Conclusions

Antibody-mediated tumour vascular targeting could constitute an attractive alternative to similar humoral approaches directed at the parenchymal component of solid tumours. Unlike tumour-associated antigens expressed by cancer cells, tumour endothelial proliferation antigens are highly accessible to circulating antibodies. The failure of a single tumour capillary as a result of this and other anti-vascular strategies could lead to the ischaemic death of many malignant cells with nutritional dependence on the targeted vessel. The development of clinical applications for the related anti-angiogenesis approach looks an increasingly realistic prospect given the plethora of agents with anti-angiogenic properties.

The targeting of tumour endothelial proliferation antigens and other anti-vascular approaches, in common with anti-angiogenesis strategies, suffers from the theoretical problem that peripheral tumour cells could survive on a diffusion-dependent basis. However, these surviving cells should be readily susceptible to conventional cancer treatments. An additional cause for concern is the effect of these approaches on wound healing, endometrial proliferation, placental development and other physiological processes involving proliferating endothelium. The theoretical benefits of vascular targeting and anti-angiogenesis strategies for cancer therapy still have to be realised in the clinical setting, but these approaches represent an attractive means of overcoming constraints imposed on conventional cancer therapy by the tumour vasculature.

### References

ABRAHAM JA, MERGIA A, WHANG JL, TUMOLO A, FRIEDMAN J, HJERRILD KA, GOSPODAROWICZ D AND FIDDES JC. (1986). Nucleotide sequence of a bovine clone encoding the angiogenic protein, basic fibroblast growth factor. *Science*, **233**, 545–548.

ALBO D, GRANICK MS, JHALA N, ATKINSON B AND SOLOMON MP. (1994). The relationship of angiogenesis to biological activity in human squamous cell carcinomas of the head and neck. *Ann. Plastic Surg.*, **32**, 588–594.

ALGIRE GH, CHALKLEY HW, LEGALLIS FY AND PARK HD. (1945). Vascular reactions of normal and malignant tissues *in vivo*. Vascular reactions of mice to wounds and to normal and neoplastic transplants. *J. Natl Cancer Inst.*, **6**, 73–85.

ALLIEGRO MC, ALLIEGRO MA, CRAGOE EJ AND GLASER BM. (1993). Amiloride inhibition of angiogenesis *in vitro*. *J. Exp. Zool.*, **267**, 245–252.

BARRIE R, WOLTERING EA, HAJARIZADEH H, MUELLER C, URE T AND FLETCHER WS. (1993). Inhibition of angiogenesis by somatostatin and somatostatin-like compounds is structurally dependent. *J. Surg. Res.*, **55**, 446–450.

BEGG AC AND TERRY NHA. (1983). Modification of stromal radiosensitivity by misonidazole and WR-2721. *Br. J. Radiol.*, **56**, 565–570.

BENEZRA D. (1978). Neovasculogenic ability of prostaglandins, growth factors and synthetic chemoattractants. *Am. J. Opthalmol.*, **86**, 455–461.

BICKNELL R AND HARRIS AL. (1991). Novel growth regulatory factors and tumour angiogenesis. *Eur. J. Cancer*, **27**, 781–785.

BICKNELL R AND HARRIS AL. (1992). Anti-cancer strategies involving the vasculature. Vascular targetting and the inhibition of angiogenesis. *Semin. Cancer Biol.*, **3**, 399–407.

BLEI F, WILSON L, MIGNATTI P AND RIFKIN DB. (1993). Mechanism of action of angiostatic steroids: suppression of plasminogen activator activity via stimulation of plasminogen activator inhibitor synthesis. *J. Cell Physiol.*, **155**, 568–578.

BLOKSMA N, HOFHUIS F, BENAISSA-TROUW B AND WILLIERS J. (1982). Endotoxin-induced release of tumour necrosis factor and interferon *in vivo* is inhibited by prior adrenoceptor blockade. *Cancer Immunol. Immunother.*, **14**, 41–45.

BOSARI S, LEE AK, DELELLIS RA, WILEY BD, HEATLEY GJ AND SILVERMAN ML. (1992). Microvessel quantitation and prognosis in invasive breast carcinoma. *Hum. Pathol.*, **23**, 755–761.

BRULAND OS, FODSTAD O, STENWIG AE AND PIHL A. (1988). Expression and characteristics of a novel human osteosarcoma-associated surface antigen. *Cancer Res.*, **48**, 5302–5309.

BUGELSKI PJ, PORTER CW AND DOUGHERTY TJ. (1981). Autoradiographic distribution on hematoporphyrin derivative in normal and tumor tissue of the mouse. *Cancer Res.*, **41**, 4606–4612.

BURROWS FJ AND THORPE PE. (1993). Eradication of large solid tumors in mice with an immunotoxin directed against tumor vasculature. *Proc. Natl Acad. Sci. USA*, **90**, 8996–9000.

BURROWS FJ, WATANABE Y AND THORPE PE. (1992). A murine model for antibody-directed targeting of vascular endothelial cells in solid tumors. *Cancer Res.*, **52**, 5954–5962.

CARMICHAEL DF, SOMMER A, THOMPSON RC, ANDERSON DC, SMITH CG, WELGUS HG AND STRICKLIN GP. (1986). Primary structure and cDNA cloning of human fibroblast collagenase inhibitor. *Proc. Natl Acad. Sci. USA*, **83**, 2407–2411.

CARSWELL EA, OLD LJ, KASSEL RL, GREEN S, FIORE N AND WILLIAMSON B. (1975). An endotoxin-induced serum factor that causes necrosis of tumours. *Proc. Natl Acad. Sci. USA*, **72**, 3666–3670.

CHEIFETZ S, BELLON T, CALES C, VERA S, BERNABEU C, MASSAGUE J AND LETARTE M. (1992). Endoglin is a component of the transforming growth factor-B receptor system in human endothelial cells. *J. Biol. Chem.*, **267**, 19027–19030.

CHODAK GW, HAUDENSCHILD C, GITTES R AND FOLKMAN J. (1980). Angiogenic activity as a marker of neoplastic and preneoplastic lesion of the human bladder. *Ann. Surg.*, **192**. 762–771.

CLAPP C, MARTIAL JA, GUZMAN RC, RENTIER-DELRUE F AND WEINER RI. (1993). The 16-kilodaton N-terminal fragment of human prolactin is a potent inhibitor of angiogenesis. *Endocrinology*, **133**, 1292–1299.

CLARKE MSF AND WEST DC. (1991). The identification of proliferation and tumour-induced proteins in human endothelial cells: a possible target for tumour therapy. *Electrophoresis*, **12**, 500–508.

CLAUSS M, MURRAY JC, VIANNA M, DE WAAL R, THURSTON G, NAWROTH P, GERLACH H, BACH R, FAMILLETTI PC AND STERN D. (1990). A polypeptide factor produced by fibrosarcoma cells that induces endothelial tissue factor and enhances the procoagulant response to tumor necrosis factor/cachectin. *J. Biol. Chem.*, **265**, 7078–7083.

DANESI R, DEL BIANCHI S, SOLDANI P, CAMPAGNI A, LA ROCCA RV, MYERS CE, PAPARELLI A AND DEL TACCA M. (1993). Suramin inhibits bFGF-induced endothelial cell proliferation and angiogenesis in the chick chorioallantoic membrane. *Br. J. Cancer*, **68**, 932–938.

DENEKAMP J. (1982). Endothelial cell proliferation as a novel approach to targeting tumour therapy. *Br. J. Cancer*, **45**, 136–139.
DENEKAMP J. (1990). Vascular attack as a therapeutic strategy for cancer. *Cancer Metastasis Rev.*, **9**, 267–282.
DENEKAMP J. (1993). Angiogenesis, neovascular proliferation and vascular pathophysiology as targets for cancer therapy. *Br. J. Radiol*, **66**, 181–196.
DENEKAMP J AND HOBSON B. (1982). Endothelial cell proliferation in experimental tumours. *Br. J. Cancer*, **46**, 711–720.
DERYNCK R, JARRETT JA, CHEN EY, EATON DH, BELL JR, ASSOIAN RK, ROBERTS AB, SPORN MB AND GOEDDEL DV. (1985). Human transforming growth factor-$\beta$ complementary DNA sequence and expression in normal and transformed cells. *Nature*, **316**, 701–705.
DVORAK HF AND GRESSER I. (1989). Microvascular injury in pathogenesis of interferon-induced necrosis of subcutaneous tumors in mice. *J. Natl Cancer Inst.*, **81**, 497–502.
DVORAK HF, NAGY JA, DVORAK JT AND DVORAK AM. (1988). Identification and characterization of the blood vessels of solid tumors that are leaky to circulating macromolecules. *Am. J. Pathol.*, **133**, 95–109.
DVORAK HF, NAGY JA AND DVORAK AM. (1991). Structure of solid tumours and their vasculature: implications for therapy with monoclonal antibodies. *Cancer Cells*, **3**, 77–85.
ENDRICH B, ZWEIFACH BW, REINHOLD HS AND INTAGLIETTA M. (1979). Quantitative studies of microcirculatory function in malignant tissue: influence of temperature on microvascular haemodynamics during the early growth of the BA1112 rat sarcoma. *Int. J. Radiat. Oncol. Biol. Phys.*, **5**, 2021–2030.
ESCH F, UENO N, BAIRD A, HILL F, DENOROY L, LING N, GOSPODAROWICZ D AND GUILLEMIN R. (1985*a*). Primary structure of bovine brain acidic fibroblast growth factor (FGF). *Biochem. Biophys. Res. Commun.*, **133**, 554–562.
ESCH F, BAIRD A, LING N, UENO N, HILL F, DENOROY L, KLEPPER R, GOSPODAROWICZ D, BOHLEN P AND GUILLEMIN R. (1985*b*). Primary structure of bovine pituitary basic fibroblast growth factor (FGF) and comparison with the amino-terminal sequence of bovine brain acidic FGF. *Proc. Natl Acad. Sci. USA*, **82**, 6507–6511.
FAJARDO LF, SCHREIBER AB, KELLY NI AND HAHN GM. (1985). Thermal sensitivity of endothelial cells. *Radiat. Res.*, **103**, 276–285.
FAN TD, HU D, GUARD S, GRESHAM GA AND WATLING KJ. (1993). Stimulation of angiogenesis by substance P and interleukin-1 in the rat and its inhibition by $NK_1$ or interleukin-1 receptor antagonists. *Br. J. Pharmacol.*, **110**, 43–49.
FANG W, HARTMANN N, CHOW DT, RIEGEL AT AND WELLSTEIN A. (1992). Pleitrophin stimulates fibroblasts and endothelial cells and epithelial cells and is expressed in human cancer. *J. Biol. Chem.*, **267**, 25889–25897.
FERNANDEZ LA, TWICKLER J AND MEAD A. (1985). Neovascularization produced by angiotensin II. *J. Lab. Clin. Med.*, **105**, 141–145.
FETT JW, STRYDOM DJ, LOBB RR, ALDERMAN EM, BETHUNE JL, RIORDAN JF AND VALLEE BL. (1985). Isolation and characterization of angiogenin, an angiogenic protein from human carcinoma cells. *Biochemistry*, **24**, 5480–5486.
FINNIS C, DODSWORTH N, POLLITT CE, CARR G AND SLEEP D. (1993). Thymidine phosphorylase activity of platelet-derived endothelial cell growth factor is responsible for endothelial cell mitogenicity. *Eur. Jo. Biochem.*, **212**, 201–210.
FOLKMAN J. (1972). Anti-angiogenesis: new concept for therapy of solid tumors. *Ann. Surg.*, **175**, 409–416.
FOLKMAN J. (1984). Angiogenesis. In *Biology of Endothelial Cells*, Jaffe EA. (ed.) pp. 412–428. Martinus Nijhoff: The Hague.
FOLKMAN J. (1985) Tumour angiogenesis. *Adv. Cancer Res.*, **43**, 175–203.
FOLKMAN J AND INGBER DE. (1987). Angiostatic steroids. *Ann. Surg.*, **206**, 374–383.
FOLKMAN J, MERLER E, ABERNATHY C AND WILLIAMS G. (1971). Isolation of a tumor factor responsible for angiogenesis. *J. Exp. Med.*, **133**, 275–288.
FOLKMAN J, WEISZ PB, JOULLIÉ MM, LI WW AND EWING WR. (1989). Control of angiogenesis with synthetic heparin substitutes. *Science*, **243**, 1490–1493.
FORM DM AND AUERBACK R. (1983). $PGE_2$ and angiogenesis. *Proc. Soc. Exp. Biol. Med.*, **172**, 214–218.
FOX SB, GATTER KC, BICKNELL R, GOING JJ, STANTON P, COOKE TG AND HARRIS AL. (1993). Relationship of endothelial cell proliferation to tumour vascularity in human breast cancer. *Cancer Res.*, **53**, 4161–4163.
FRATER-SCHRODER M, RISAU W, HALLMANN R, GAUTSCHI P AND BOHLEN P. (1987). Tumor necrosis factor type $\alpha$, a potent inhibitor of endothelial cell growth *in vitro*, is angiogenic *in vivo*. *Proc. Natl Acad. Sci. USA*, **84**, 5277–5281.
FREGENE TA, KHANUJA PS, NOTO AC, GEHANI SK, VAN EGMONT EM, LUZ DA AND PIENTA KJ. (1993). Tumor-associated angiogenesis in prostatic cancer. *Anticancer Res.*, **13**, 2377–2381.
FREUDENBERG N, RIESE KH AND FREUDENBERG MA. (1983). The vascular endothelial system. *Veroff Pathol.*, **120**, 1–114.
FURUKAWA T, YOSHIMURA A, SUMIZAWA T, HARAGUCHI M AND AKIYAMA S. (1992). Angiogenic factor. *Nature*, **356**, 668.
GIMBRONE MA, COTRAN RS, LEAPMAN SB AND FOLKMAN J. (1974). Tumor growth and neovascularization: an experimental model using the rabbit cornea. *J. Natl Cancer Inst.*, **52**, 413–427.
GIMBRONE MA, LEAPMAN SB, COTRAN RS AND FOLKMAN J. (1972). Tumor dormancy *in vivo* by prevention of neovascularization. *J. Exp. Med.*, **136**, 261–276.
GOOD DJ, POLVERINI PJ, RASTINEJAD F, LE BEAU MM, LEMONS RS, FRAZIER WA AND BOUCK NP. (1990). A tumor supressor-dependent inhibitor of angiogenesis is immunologically and functionally indistinguishable from a fragment of thrombospondin. *Proc. Natl Acad. Sci. USA*, **87**, 6624–6628.
GOUGOS A AND LETARTE M. (1990). Primary structure of endoglin, an RGD-containing glycoprotein of human endothelial cells. *J. Biol. Chem.*, **265**, 8361–8364.
GRANT DS, TASHIRO K, SEGUI-REAL B, YAMADA Y, MARTIN GR AND KLEINMAN HK. (1989). Two different laminin domains mediate the differentiation of human endothelial cells into capillary-like structures *in vitro*. *Cell*, **58**, 933–943.
GROSS JL, HERBLIN WF, DUSAK BA, CZERNIAK P, DIAMOND M AND DEXTER DL. (1990). Modulation of solid tumor growth *in vivo* by bFGF (abstract). *Proc. Am. Assoc. Cancer Res.*, **31**, 79.
GUNDUZ N. (1981). Cytokinetics of tumour and endothelial cells and vascularization of lung metastases in C3H/He mice. *Cell Tissue Kinetics*, **14**, 343–363.
HAGEMEIER H, VOLLMER E, GOERDT S, SCHULZE-OSTHOFF K AND SORG C. (1986). A monoclonal antibody reacting with endothelial cells of budding vessels in tumors and inflammatory tissues, and non-reactive with normal adult tissues. *Int. J. Cancer*, **38**, 481–488.
HART MN, DEBAULT LE, SADEWASSER KL, CANCILLA PA AND HENRIQUEZ EM. (1981). Morphological effects of antibody to mouse brain endothelium *in vivo*. *J. Neuropathol. Exp. Neurol.*, **40**, 84–91.
HERTIG AT. (1935). Angiogenesis in the early human chorion and in the primary placenta of the Macaque monkey. *Contrib. Embryol.*, **25**, 37–81.
HILLS S, WILLIAMS KB AND DENEKAMP J. (1989). Vascular collapse after flavone acetic acid: a possible mechanism for its anti-tumour action. *Eur. J. Cancer Clinical Oncol.*, **25**, 1419–1424.
HIRST DG, DENEKAMP J AND HOBSON B. (1982). Proliferation kinetics of endothelial and tumour cells in three mouse mammary carcinomas. *Cell Tissue Kinetics*, **15**, 251–261.
HORAK ER, LEEK R, DELELLIS RA, WILEY BC, HEATLEY GJ AND SILVERMAN ML. (1992). Quantitative angiogenesis assessed by anti-PECAM (platelet/endothelial cell adhesion molecule) antibodies: correlation with node metastases and survival in breast cancer. *Lancet*, **340**, 1120–1124.
HORI A, SASADA R, MATSUTANI E, NAITO K, SAKURA Y, FUJITA T AND KOZAI Y. (1991). Suppression of solid tumor growth by immunoneutralizing monoclonal antibody against human basic fibroblast growth factor. *Cancer Res.*, **51**, 6180–6184.
HORI K, SUZUKI M, TANDA S AND SAITO S. (1990). *In vivo* analysis of tumor vascularization in the rat. *Jpn J. Cancer Res.*, **81**, 279–288.
IDE AG, BAKER NH AND WARREN SL. (1939). Vascularization of the Brown–Pearce rabbit epithelioma transplant as seen in the transparent ear chamber. *Am. J. Roentgenol.*, **42**, 891–899.
INGBER D. (1991). Extracellular matrix and cell shape: potential control points for inhibition of angiogenesis. *J. Cell Biochem.*, **47**, 236–241.
INGBER DE AND FOLKMAN J. (1989). How does extracellular matrix control capillary morphogenesis? *Cell*, **58**, 803–805.
INGBER D, FUJITA T, KISHIMOTO S, SUDO K, KANAMARU T, BREM H AND FOLKMAN J. (1990). Synthetic analogues of fumagillin that inhibit angiogenesis and suppress tumour growth. *Nature*, **348**, 555–557.

ISHIKAWA F, MIYAZONO K, HELLMAN U, DREXLER H, WERNSTEDT C, HAGIWARA K, USUKI K, TAKAKU F, RISAU W AND HELDIN C-H. (1989). Identification of angiogenic activity and the cloning and expression of platelet-derived endothelial cell growth factor. *Nature*, **338**, 557-561.

KALLINOWSKI F, SCHAEFER C, TYLER G AND VAUPEL P. (1989). *In vivo* targets of recombinant tumour necrosis factor-α: blood flow, oxygen consumption and growth of isotransplanted rat tumours. *Br. J. Cancer*, **60**, 555-560.

KAO J, RYAN J, BRETT G, CHEN J, SHEN H, FAN YG, GODMAN G, FAMILLETTI PC, WANG F, PAN YC, STERN D AND CLAUSS M. (1992). Endothelial monocyte-activating polypeptide II. A novel tumor-derived polypeptide that activates host-response mechanisms. *J. Biol. Chem.*, **267**, 20239-20247.

KECK PJ, HAUSER SD, KRIVI G, SANZO K, WARREN T, FEDER J AND CONNOLLY DT. (1989). Vascular permeability factor, an endothelial cell mitogen related to PDGF. *Science*, **246**, 1309-1311.

KIM KJ, LI B, WINER J, ARMANINI M, GILLETT N, PHILLIPS HS AND FERRARA N. (1993). Inhibition of vascular endothelial growth factor-induced angiogenesis suppresses tumour growth *in vivo*. *Nature*, **362**, 841-844.

KLAGSBRUN M AND D'AMORE PA. (1991). Regulators of angiogenesis. *Annu. Rev. Physiol.*, **53**, 217-239.

KNIGHTON D, AUSPRUNK D, TAPPER D AND FOLKMAN J. (1977). Avascular and vascular phases of tumour growth in the chick embryo. *Br. J. Cancer*, **35**, 347-356.

KONDO S, ASANO M AND SUZUKI H. (1993). Significance of vascular endothelial growth factor/vascular permeability factor for solid tumor growth, and its inhibition by the antibody. *Biochem. Biophys. Res. Commun.*, **194**, 1234-1241.

KOTASEK D, VERCELLOTTI GM, OCHOA AC, BACH FH, WHITE JG AND JACOB HS. (1988). Mechanism of cultured endothelial injury induced by lymphokine-activated killer cells. *Cancer Res.*, **48**, 5528-5532.

KULL JR FC, BRENT DA, PARIKH I AND CUATRECASAS P. (1987). Chemical identification of a tumor-derived angiogenic factor. *Science*, **236**, 843-845.

KURACHI K, DAVIE EW, STRYDOM DJ, RIORDAN JF AND VALLEE BL. (1985). Sequence of the cDNA and gene for angiogenin, a human angiogenesis factor. *Biochemistry*, **24**, 5494-5499.

LASTRES P, BELLON T, CABANAS C, SANCHEZ-MADRID F, ACEVEDO A, GOUGOS A, LETARTE M AND BERNABEU C. (1992). Regulated expression on human macrophages of endoglin, an Arg-Gly-Asp-containing surface antigen. *Eur. J. Immunol.*, **22**, 393-397.

LAZO JS. (1986). Endothelial injury caused by antineoplastic agents. *Biochem. Pharmacol.*, **35**, 1919-1923.

LEIBOVICH SJ, POLVERINI PJ, SHEPARD HM, WISEMAN DM, SHIVELY V AND NUSEIR N. (1987). Macrophage-induced angiogenesis is mediated by tumour necrosis factor-α. *Nature*, **329**, 630-632.

LEONE A, FLATOW U, KING CR, SANDEEN MA, MARGULIES IM, LIOTTA LA AND STEEG PS. (1991). Reduced tumor incidence, metastatic potential and cytokine responsiveness of nm23-transfected melanoma cells. *Cell*, **65**, 25-35.

LEUNG DW, CACHIANES G, KUANG W, GOEDDEL DV AND FERRARA N. (1989). Vascular endothelial growth factor is a secreted angiogenic mitogen. *Science*, **246**, 1306-1309.

LIEN WM AND ACKERMAN NB. (1970). The blood supply of experimental liver metastases. II. A microcirculatory study of normal and tumor vessels of the liver with the use of perfused silicone rubber. *Surgery*, **68**, 334-340.

LIOTTA LA, STEEG PS AND STETLER-STEVENSON WG. (1991). Cancer metastasis and angiogenesis: an imbalance of positive and negative regulation. *Cell*, **64**, 337-350.

MCAUSLAN BR AND HOFFMAN H. (1979). Endothelium stimulating factor from Walker carcinoma cells. *Exp. Cell Res.*, **119**, 181-190.

MACCHIARINI P, FONTANINI G, HARDIN MJ, SQUARTINI F AND ANGELETTI CA. (1992). Relation of neovascularization to metastasis of non-small-cell lung cancer. *Lancet*, **340**, 145-146.

MAIONE TE, GRAY GS, PETRO J, HUNT AJ, DONNER AL, BAUER SI, CARSON HF AND SHARPE RJ. (1990). Inhibition of angiogenesis by recombinant human platelet factor-4 and related peptides. *Science*, **247**, 77-79.

MARQUARDT H, HUNKAPILLER MW, HOOD LE AND TODARO GJ. (1984). Rat transforming growth factor type 1: structure and relation to epidermal growth factor. *Science*, **233**, 1079-1082.

MATSUBARA T, SAURA R, HIROHATA K AND ZIFF M. (1989). Inhibition of human endothelial cell proliferation *in vitro* and neovascularization *in vivo* by D-penicillamine. *J. Clin. Invest.*, **83**, 158-167.

MATSUZAKI K, YOSHITAKE Y, MATUO Y, SASAKI H AND NISHIKAWA K. (1989). Monoclonal antibodies against heparin-binding growth factor II/basic fibroblast growth factor that block its biological activity: Invalidity of the antibodies for tumor angiogenesis. *Proc. Natl Acad. Sci. USA*, **86**, 9911-9915.

MATTSSON J, APPELGREN L, HAMBERGER B AND PETERSON H. (1979). Tumor vessel innervation and influence of vasoactive drugs on tumor blood flow. In *Tumor Blood Circulation: Angiogenesis, Vascular Morphology and Blood Flow of Experimental and Human Tumors*, Peterson H. (ed.) pp. 129-135. CRC Press: Boca Raton, FL.

MILLAUER B, SHAWVER LK, PLATE KH, RISAU W AND ULLRICH A. (1994). Glioblastoma growth inhibited *in vivo* by a dominant-negative Flk-1 mutant. *Nature*, **367**, 576-579.

MOGHADDAM A AND BICKNELL R. (1992). Expression of platelet-derived endothelial cell growth factor in *Escherichia coli* and confirmation of its thymidine phosphorylase activity. *Biochemistry*, **31**, 12141-12146.

MORRIS PB, ELLIS MN AND SWAIN JL. (1989). Angiogenic potency of nucleotide metabolites: potential role in ischaemia-induced vascular growth. *J. Mol. Cell Cardiol.*, **21**, 351-358.

MOSES MA, SUDHALTER J AND LANGER R. (1990). Identification of an inhibitor of neovascularization from cartilage. *Science*, **248**, 1408-1410.

MOTRO B, ITIN A, SACHS L AND KESHET E. (1990). Pattern of interleukin 6 gene expression *in vivo* suggests a role for this cytokine in angiogenesis. *Proc. Natl Acad. Sci. USA*, **87**, 3092-3096.

MULLER WA, RATTI CM, McDONNELL SL AND COHN ZA. (1989). A human endothelial cell-restricted, externally disposed plasmalemmal protein enriched in intercellular junctions. *J. Exp. Med.*, **170**, 399-340.

MURATA J, SAIKI I, MAKABE T, TSUTA Y, TOKURA S AND AZUMA I. (1991). Inhibition of tumor-induced angiogenesis by sulfated chitin derivatives. *Cancer Res.*, **51**, 22-26.

MURRAY JC, RANDHAWA VS AND DENEKAMP J. (1987). The effects of melphalan and misonidazole on the vasculature of murine sarcoma. *Br. J. Cancer*, **55**, 233-238.

MURRAY JC, SMITH KA AND LAUK S. (1989). Vascular markers for murine tumours. *Radiother. Oncol.*, **16**, 221-234.

ODEDRA R AND WEISS JB. (1991). Low molecular weight angiogenesis factors. *Pharmacol. Ther.*, **49**, 111-124.

OHTSU A, FUJII K AND KUROZUMI S. (1988). Induction of angiogenic response by chemically stable prostacyclin analogs. *Prostaglandins Leukotines Essential Fatty Acids*, **33**, 35-39.

OIKAWA T, HIROTANI K, SHIMAMURA M, ASHINO-FUSE H AND IWAGUCHI T. (1989). Powerful antiangiogenic activity of herbimycin A (named angiostatic antibiotic). *J. Antibiot.*, **42**, 1202-1204.

OIKAWA T, HIROTANI K, OGASAWARA H, KATAYAMA T, NAKAMURA O, IWAGUCHI T AND HIRAGUN A. (1990). Inhibition of angiogenesis by vitamin $D_3$ analogues. *Eur. J. Pharmacol.*, **178**, 247-250.

OIKAWA T, SHIMAMURA M, ASHINO-FUSE H, IWAGUCHI T, ISHIZUKA M AND TAKEUCHI T. (1991). Inhibition of angiogenesis by 15-deoxyspergualin. *J. Antibiot.*, **44**, 1033-1035.

OOSTERWIJK E, RUITER DJ, WAKKA JC, HUISKENS-VAN DER MEIJ JW, JONAS U, FLEUREN GJ, ZWARTENDIJK J, HOEDEMAEKER P AND WARNAAR SO. (1986). Immunohistochemical analysis of monoclonal antibodies to renal antigens: application in the diagnosis of renal cell carcinoma. *Am. J. Pathol.*, **123**, 301-309.

ORCHARD PJ, SMITH CM, WOODS WG, DAY DL, DEHNER LP AND SHAPIRO R. (1989). Treatment of haemangioendotheliomas with alpha interferon. *Lancet*, **8662**, 565-567.

PHILLIPS P AND KUMAR S. (1979). Tumour angiogenesis factor (TAF) and its neutralization by a xenogenic antiserum. *Int. J. Cancer*, **23**, 82-88.

PLATE KH, BREIER G, WEICH HA AND RISAU W. (1992). Vascular endothelial growth factor is a potent tumour angiogenesis factor in human gliomas *in vivo*. *Nature*, **359**, 845-848.

RASTINEJAD F, POLVERINI PJ AND BOUCK NP. (1989). Regulation of the activity of a new inhibitor of angiogenesis by a cancer suppressor gene. *Cell*, **56**, 345-355.

REILLY TM, TAYLOR DS, HERBLIN WF, THOOLEN MJ, CHIU AT, WATSON DW AND TIMMERMANS PB. (1989). Monoclonal antibodies directed against basic fibroblast growth factor which inhibit its biological activity *in vitro* and *in vivo*. *Biochem. Biophys. Res. Commun.*, **164**, 736-743.

REINHOLDS HS, BLACHIWIECZ B AND VAN DEN BERG-BLOK AE. (1978). *Cancer Therapy by Hyperthermia and Radiation.* Urban and Schwarzenberg: Baltimore.

RETTIG WJ, GARIN-CHESA P, HEALEY JH, SU SL, JAFFE EA AND OLD LJ. (1992). Identification of endosialin, a cell surface glycoprotein of vascular endothelial cells in human cancer. *Proc. Natl Acad. Sci. USA*, **89**, 10832-10836.

RISAU W, DREXLER H, MIRONOV V, SMITS A, SIEGBAHN A, FUNA K AND HELDIN CH. (1992). Platelet-derived growth factor is angiogenic *in vivo*. *Growth Factors*, **7**, 261-266.

ROBERTS AB, SPORN MB, ASSOIAN RK, SMITH JM, ROCHE NS, WAKEFIELD LM, HEINE UI, LIOTTA LA, FALANGA V, KEHRL JH AND FAUCI AS. (1986). Transforming growth factor type $\beta$: rapid induction of fibrosis and angiogenesis *in vivo* and stimulation of collagen formation *in vitro*. *Proc. Natl Acad. Sci. USA*, **83**, 4167-4171.

ROSENGARD AM, KRUTZSCH HC, SHEARN A, BIGGS JR, BARKER E, MARGULIES IM, KING CR, LIOTTA LA AND STEEG PS. (1989). Reduced nm23/awd protein in tumour metastasis and aberrant *Drosophila* development. *Nature*, **342**, 177-180.

SCHREIBER AB, WINKLER ME AND DERYNCK R. (1986). Transforming growth factor-$\alpha$: a more potent angiogenic mediator than epidermal growth factor. *Science*, **232**, 1250-1253.

SCHULTZ-HECTOR S AND HAGHAYEGH S. (1993). $\beta$-Fibroblast growth factor expression in human and murine squamous cell carcinomas and its relationship to regional endothelial proliferation. *Cancer Res.*, **53**, 1444-1449.

SENGER DR, CONNOLLY DT, VAN DE WATER L, FEDER J AND DVORAK HF. (1990). Purification and $NH_2$-terminal amino acid sequence of guinea pig tumor-secreted vascular permeability factor. *Cancer Res.*, **50**, 1774-1778.

SHAPIRO R AND VALLEE BL. (1987). Human placental ribonuclease inhibitor abolishes both angiogenic and ribonucleolytic activities of angiogenin. *Proc. Natl Acad. Sci. USA*, **84**, 2238-2241.

SHWEIKI D, ITIN A, SOFFER D AND KESHET E. (1992). Vascular endothelial growth factor induced by hypoxia may mediate hypoxia-initiated angiogenesis. *Nature*, **359**, 843-845.

SIDKY YA AND BORDEN EC. (1987). Inhibition of angiogenesis by interferons: effects on tumor- and lymphocyte-induced vascular responses. *Cancer Res.*, **47**, 5155-5161.

SOLESVIK OV, ROFSTAD EK AND BRUSTAD T. (1984). Vascular changes in a human malignant melanoma xenograft following single-dose irradiation. *Radiat Res.*, **98**, 115-128.

SRIVASTAVA A, LAIDLER P, DAVIES RP, MORGAN K AND HUGHES LE. (1988). The prognostic significance of tumor vascularity in intermediate-thickness (0.74-4.0 mm thick) skin melanoma. *Am. J. Pathol.*, **133**, 419-423.

STAR WM, MARIJNISSEN HPA, VAN DEN BERG-BLOK AE, VERSTEEG JAC, FRANKEN KAP AND REINHOLD HS. (1986). Destruction of rat mammary tumor and abnormal tissue microcirculation by haematoporphyrin derivative photoradiation observed *in vivo* in sandwich observation chambers. *Cancer Res.*, **46**, 2531-2540.

STETLER-STEVENSON WG, KRUTZSCH HC AND LIOTTA LA. (1989). Tissue inhibitor of metalloproteinase (TIMP-2). *J. Biol. Chem.*, **264**, 17374-17378.

TAMARGO RJ, BOK RA AND BREM H. (1991). Angiogenesis inhibition by minocycline. *Cancer Res.*, **51**, 672-675.

TANNOCK IF. (1970). Population kinetics of carcinoma cells, capillary endothelial cells, and fibroblasts in a transplanted mouse mammary tumor. *Cancer Res.*, **30**, 2470-2476.

TAYLOR S AND FOLKMAN J. (1982). Protamine is an inhibitor of angiogenesis. *Nature*, **297**, 307-312.

THOMLINSON RH AND GRAY LH. (1965). The histological structure of some human lung cancers and the possible implications for radiotherapy. *Br. J. Cancer*, **9**, 539-549.

THORPE PE, DERBYSHIRE EJ, KING SW AND BURROWS FJ. (1994). Targeting the vasculature of carcinomas and other solid tumors (abstract). *Am. Assoc. Cancer Res.* **35**, 379.

USUKI K, SARAS J, WALTENBERGER J, MIYAZONO K, PIERCE G, THOMASON A AND HELDIN CH. (1992). Platelet-derived endothelial cell growth factor has thymidine phosphorylase activity. *Biochem. Biophys. Res. Commun.*, **184**, 1311-1316.

VAUPEL P, KALLINOWSKI F AND OKUNIEFF P. (1989). Blood flow, oxygen and nutrient supply, and metabolic microenvironment of human tumors: a review. *Cancer Res.*, **49**, 6449-6465.

VUKANOVIC J, PASSANITI A, HIRATA T, TRAYSTMAN RJ, HARTLEY-ASP B AND ISAACS JT. (1993). Antiangiogenic effects of the quinoline-3-carboxamide linomide. *Cancer Res.*, **53**, 1833-1837.

WAKAMATSU K, MASAKI T, ITOH F, KONDO K AND SUDO K. (1990). Isolation of a fatty acid amide as an angiogenic principle from bovine mesentery. *Biochem. Biophys. Res. Commun.*, **168**, 423-429.

WANG JM, KUMAR S, PYE D, VAN AGTHOVEN AJ, KRUPINSKI J AND HUNTER RD. (1993). A monoclonal antibody detects heterogeneity in vascular endothelium of tumours and normal tissues (abstract). *Int. J. Cancer*, **54**, 363-370.

WARD WF, SOLLIDAY NH, MOLTENI A AND PORT CD. (1983). Radiation injury in rat lung II. Angiotensin-converting enzyme activity. *Radiat Res.*, **96**, 294-300.

WARREN BA. (1979). The vascular morphology of tumors. In *Tumor Blood Circulation: Angiogenesis, Vascular Morphology and Blood Flow of Experimental and Human Tumors*, Peterson H. (ed.) pp. 1-47. CRC Press: Boca Raton, FL.

WEIDNER N. (1993). Tumor angiogenesis: review of current applications in tumor prognostication. *Semin. Diagnostic Pathol.*, **10**, 302-313.

WEIDNER N, SEMPLE JP, WELCH WR AND FOLKMAN J. (1991). Tumor angiogenesis and metastasis - correlation in invasive breast carcinoma. *N. Engl. J. Med.*, **32**, 1-8.

WEIDNER N, FOLKMAN J, POZZA F, BEVILACQUA P, ALLRED EN, MOORE DH, MELI S AND GASPARINI G. (1992). Tumor angiogenesis: a new significant and independent prognostic indicator in early-stage breast carcinoma. *J. Natl Cancer Inst.*, **84**, 1875-1887.

WEISS JB, BROWN RA, KUMAR S AND PHILLIPS P. (1979). An angiogenic factor isolated from tumours: a potent low-molecular-weight compound. *Br. J. Cancer*, **40**, 493-496.

WEISS RB, GREENE RF, KNIGHT RD, COLLINS JM, PELOSI JJ, SULKES A AND CURT GA. (1988). Phase 1 and clinical pharmacology study of intravenous flavone acetic acid (NSC 347512). *Cancer Res.*, **48**, 5878-5882.

WEST CML, WEST DC AND KUMAR S. (1990). A comparison of the sensitivity to photodynamic treatment of endothelial and tumour cells in different proliferative states. *Int. J. Radiat. Biol.*, **58**, 145-156.

YATES RA, NANNEY LB, GATES RE AND KING JR LE. (1991). Epidermal growth factor and related growth factors. *Int. J. Dermatol.*, **30**, 687-694.

ZICHE M AND GULLINO PM. (1982). Angiogenesis and neoplastic progression in vitro. *J. Natl Cancer Inst.*, **69**, 483-487.

ZICHE M, MORBIDELLI L, PACINI M, GEPPETTI P, ALESSANDRI G AND MAGGI CA. (1990). Substance P stimulates neovascularization in vivo and proliferation of cultured endothelial cells. *Microvasc. Res.*, **40**, 264-278.

ZWI LJ, BAGULEY BC, GAVIN JB AND WILSON WR. (1990). The use of vascularised spheroids to investigate the action of flavone acetic acid on tumour blood vessels. *Br. J. Cancer*, **62**, 231-237.